# Barriers and facilitators of adherence to treatment interventions for COPD amongst individuals from minority ethnic communities: Meta-ethnography

**DOI:** 10.1371/journal.pone.0318709

**Published:** 2025-02-10

**Authors:** Sarah Alamer, Anna Robinson-Barellla, Matthew Cooper, Hamde Nazar, Andy Husband

**Affiliations:** 1 School of Pharmacy, Newcastle University, Newcastle upon Tyne, United Kingdom; 2 College of Clinical Pharmacy, King Faisal University, Al-Ahsa, Saudi Arabia; 3 Population Health Sciences Institute, Newcastle University, Newcastle upon Tyne, United Kingdom; 4 Newcastle Patient Safety Research Collaboration, Newcastle University, Newcastle upon Tyne, United Kingdom; The Hong Kong Polytechnic University, HONG KONG

## Abstract

**Objective:**

Numerous studies have documented the low adherence rate to treatment interventions for Chronic Obstructive Pulmonary Disease (COPD) amongst minority ethnic communities. This systematic meta-ethnographic review was performed to identify barriers and facilitators of adherence to treatment interventions (*e*.*g*., smoking cessation and pulmonary rehabilitation) of COPD in minority ethnic communities.

**Method:**

This systematic meta-ethnographic review followed the approach by Noblit and Hare. Systematic searches were performed across six databases MEDLINE (OVID), EMBASE (OVID), CINAHL (EBSCO), PsycINFO (OVID), Web of Science, and Scopus from their inception until November 2023. Quality appraisal of included studies was conducted using Critical Appraisal Skills Programme (CASP) tools.

**Results:**

Out of 1,329 identified citations, seven qualitative studies were included in this meta-ethnography. Using reciprocal translation, four overarching themes were developed to represent the barriers and facilitators of adherence to treatment interventions of COPD amongst minority ethnic communities: 1) positive and negative experiences affecting a person’s motivation for their care, 2) patient attitude and beliefs, 3) being able to access and attend care, and 4) the influence of communication and culture on a person’s care.

**Conclusion:**

This review highlighted the barriers and facilitators of adherence to treatment interventions for COPD amongst individuals from minority ethnic communities. The key barriers include language difficulties and the inability to comprehend and communicate effectively with healthcare professionals. This demonstrates the need for further research on the impact of linguistic and cultural characteristics on adherence to treatment interventions for COPD. Addressing ethnicity-specific barriers could inform the development of tailored protocols and strategies for optimising adherence among minority ethnic communities. (PROSPERO CRD42023476187).

## Introduction

Chronic Obstructive Pulmonary Disease (COPD) is a condition that constitutes a spectrum of lung diseases, including bronchitis and emphysema, which cause respiratory symptoms such as dyspnoea, cough, and excess sputum production [1]. COPD is the fourth leading cause of death worldwide, with around 3 million deaths annually [1,2]. In the United Kingdom (UK), approximately 3 million people suffer from COPD, and it is considered the second leading cause of hospital admissions [2]. According to the Global Initiative for Obstructive Lung Disease organisation (and the definition of intervention used for this review), treatment interventions can be focused on smoking cessation, influenza vaccination, referral to and uptake of pulmonary rehabilitation, long-term oxygen therapy (LTOT), and adherence to medication, which aims to improve respiratory function [1]. Adherence to treatment interventions of COPD is crucial to enhance symptom control, reduce the risk of exacerbation and hospitalisation, and improve quality of life [1].

Variations in COPD prevalence by ethnicity have been observed globally across several countries as the United States (US) and the UK [3–6]. Within the UK population, COPD was identified to be more prevalent among people who are White when compared to minority ethnic communities in London, particularly, people from Black and South Asian ethnicities.[4,5] The term ‘minority ethnic communities’ is defined as ‘any populations that are numerically, and have different ethnic, religious, or linguistic characteristics compared to the majority population of the country where the study was conducted’ [7].

One explanation for this difference in COPD prevalence between ethnic communities is that smoking rates are reported to be higher amongst people of White ethnicity compared with other ethnic communities such as South Asian [5]. As tobacco smoking is known to be the primary cause of COPD, this disparity in smoking rates may contribute to the observed differences in disease prevalence [5]. Additionally, research has also suggested the disparity may be attributed to delayed diagnosis or inability to detect the disease within minority ethnic communities, which is a consequence of limited access to primary care [4]. Despite this, greater levels of dyspnoea (difficulty breathing or shortness of breath) and higher susceptibility to COPD exacerbations were reported amongst people from minority ethnic communities compared to those from White ethnicity [6,8]. Possible contributing factors to worsening symptoms among minority ethnic communities include poor adherence and utilisation of medical care, and limited access to healthcare resources [6]. Evidence has demonstrated associations between lower rates of adherence to treatment interventions of COPD and an individuals’ ethnicity, with the uptake of influenza vaccine, LTOT, and the application of smoking cessation programmes [9]. As a consequence, this can result in less effective disease management [1,9].

Even though treatment interventions for COPD are crucial to optimise health outcomes, several earlier studies revealed high rates of non-adherence to these interventions [1,10–12]. Prior work conducted by this research team has highlighted how ethnicity influences adherence to treatment interventions for COPD and reported lower adherence rates amongst minority ethnic communities within different countries, including the UK, US, Denmark, New Zealand, Canada, and Australia [13].

Exploring and identifying patient-related barriers and enablers influencing adherence to treatment interventions for COPD are pivotal in optimising disease outcomes among individuals from minority ethnic communities. Numerous qualitative studies have explored the barriers to treatment interventions for COPD such as smoking cessation, medication adherence, and pulmonary rehabilitation among individuals living with COPD [14–17]. However, limited research has specifically investigated the experience of people from minority ethnic communities with adherence to treatment interventions for COPD. Therefore, the purpose of this review was to explore the experiences of individuals from minority ethnic communities who have been diagnosed with COPD.

## Methods

This meta-ethnographic systematic review was conducted and reported using the meta-ethnography reporting guidance (eMERGe) and framework (S1 Table) [18] and follows a registered protocol (PROSPERO CRD42023476187).

### Search strategy and information source

A comprehensive systematic search was conducted across six electronic databases: MEDLINE (OVID), EMBASE (OVID), CINAHL (EBSCO), PsycINFO (OVID), Web of Science, and Scopus from their inception until November 2023. The search strategy was developed with assistance of an expert librarian. No restriction on publication date was applied. Forwards and backwards reference screening was performed, as well as searching grey literature (using Google Scholar and OpenGrey), to identify additional relevant papers. A full search strategy with key terms is included in the (S2 Table).

### Eligibility criteria

The Sample, Phenomenon of Interest, Design, Evaluation, and Research Type (SPIDER) tool was used to frame the inclusion criteria [19].

***Sample*:** People living with COPD who are from minority ethnic communities irrespective of their age, disease stage, and the presence of comorbidities.

***Phenomenon of Interest*:** Uptake and adherence to treatment interventions of COPD including medication taking, pulmonary rehabilitation, smoking cessation, influenza vaccination, and use of long-term oxygen therapy (LTOT).

***Design*:** Qualitative studies or mixed-method studies that included perspectives of people living with COPD towards treatment interventions (*i*.*e*., patient interviews or focus groups). Mixed-method studies will be eligible for inclusion only if sufficient qualitative data is provided in the publication or can be obtained from the authors. This review followed the seven steps of meta-ethnography, that was originally developed by Noblit and Hare [20]. These included: (1) *getting started*, (2) *deciding what is relevant to the initial interest*, (3) *reading the studies*, (4) *determining how studies are related*, (5) *translating the studies into one another*, (6) *synthesising translations*, and (7) *expressing the synthesis*.

***Evaluation*:** Studies that investigated barriers to and/or enablers of adherence to treatment interventions of COPD amongst minority ethnic communities. Studies that examined variables such as physical activity, diet, exercise training, and maintenance without focusing on adherence to treatment interventions for COPD were excluded.

***Research type*:** This meta-ethnography, as a form of qualitative evidence synthesis, only includes qualitative research. Where views of individuals from minority ethnic communities could not be independently extracted from that of other data reported, or corresponding authors could not provide data for the independent groups, the study was excluded. Additionally, Quantitative studies, systematic reviews, clinical trials, protocols, and conference abstracts were not considered. Papers written in languages other than English were also excluded due to the unavailability of translation services and time constraints within the scope of this project.

### Study selection and screening

All identified articles were exported into EndNote 20.6 for initial screening, and to detect duplicates [21]. The titles and abstracts were initially screened by one reviewer (SA) according to the inclusion criteria. Eligible articles were then imported into Rayyan software [22] and distributed among five reviewers (SA, AKH, HN, AR-B, and MC) for independent full-text assessment. No disagreements arose between the authors.

### Reading, data extraction, and quality appraisal

Two reviewers (SA and AR-B) carefully read the included studies to ensure familiarity with the data. A customised data extraction form was designed and used for data extraction across the included studies. Data extraction included information about the study and author details, country setting, study aims, participant demographics (age and sex), type of intervention, and study findings, including the main themes, original quotes, and/or explanations provided by the authors of primary studies. Quality appraisal was performed independently by SA and AR-B using the Critical Appraisal Skills Programme (CASP) for qualitative research [23]. No papers were excluded based on quality.

### Analysis and interpretive synthesis

Meta-ethnography has begun to be used more widely in healthcare research, particularly when the researcher aims to conceptualise deeper understandings of patient experiences of certain illnesses and disease-management [24]. A meta-ethnographic approach, as a form of qualitative evidence synthesis, was chosen to understand the effect of ethnicity on adherence to treatment and to develop a concept that explains the barriers and facilitators of adherence to treatment interventions of COPD amongst individuals from minority ethnic communities.

*Determining how studies are related*. A table summarising the findings extracted from the primary studies was created, incorporating the concepts and metaphors developed by the original authors. This approach facilitated the exploration and comparison of the findings. Two reviewers (SA and AR-B) then revised how the concepts and metaphors are related through discussion (*Step 4*).

*Translating the studies into one another*. Reciprocal translation was used to develop third-order constructs. This approach involves a comparison of concepts and themes from study 1 to those extracted from study 2, then comparing the combined themes from these studies, with findings from study 3, and so on. This process continued until all included papers were synthesised, and the reviewers identified and grouped similar themes. Two reviewers (SA and AR-B) were independently involved in the translation phase; no disagreements arose between the two authors. *(Step5)*

*Synthesising translations and expressing the synthesis*. Third-order constructs (overall interpretations) were developed by carefully synthesising and analysing first-order constructs (*i*.*e*., participant quotations) and the second-order constructs (*i*.*e*., original study authors’ interpretations) (*Steps 6 and 7*). To adhere to recommendations for conducting meta-ethnographies, the authors of this study use the term ‘theme’ to describe the third-order construct, and sub-themes to describe third-order construct sub-themes [18]. The development of these overarching themes enables meta-ethnographies to delve further into a topic than a traditional systematic review and contribute new insights to literature [25].

## Results

**Fig 1:**
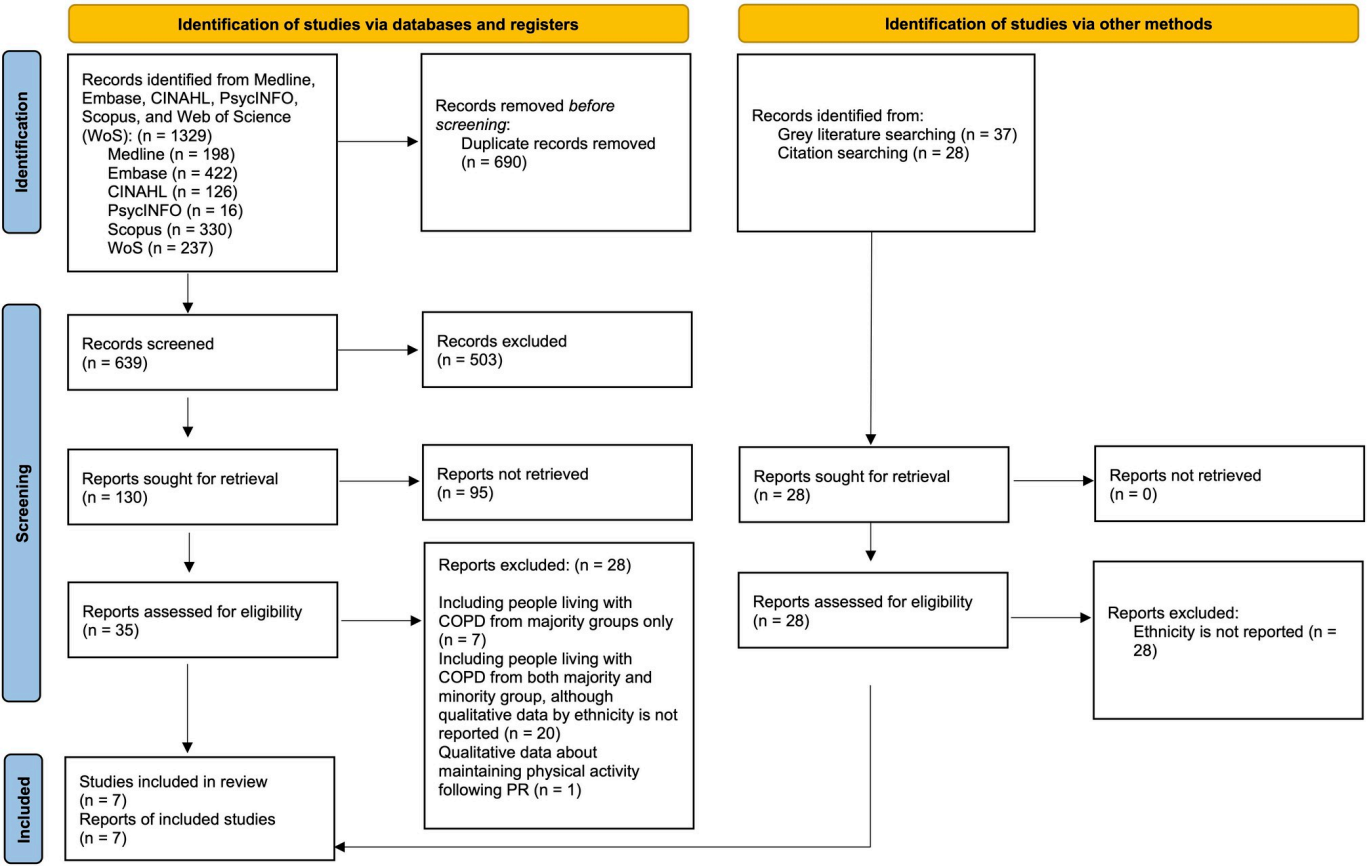
PRISMA (Preferred Reporting Items for Systematic Reviews and Meta-Analysis) flow diagram of included studies.

A total of 1,329 articles were extracted from databases and an additional 65 were identified through other sources (grey literature and the manual screening of reference lists of included articles). Once duplicates were detected and removed (n = 690), titles and abstracts of 639 articles were screened for eligibility (S3 Table). Of these, 35 articles were considered for further full-text assessment, while 28 articles were excluded due to a variety of reasons outlined in the PRISMA flowchart. (Fig 1) The remaining seven articles were eligible for inclusion in this meta-ethnographic systematic review.

### Study characteristics

The publication dates of the included articles ranged between 2015 [26] to 2023 [27]. The studies were conducted in four countries; the US (n = 3) [27–29], the UK (n = 2) [30,31], Canada (n = 1) [26], and New Zealand (n = 1) [32]. Included articles reported various minority ethnic communities including Asian [30,31], Black [30], Māori [32], Hispanic [27–29], African American [27–29], and people from Chinese ethnicity living in Canada [26]. Three types of interventions for COPD treatment were explored by the included articles; pulmonary rehabilitation (n = 5), [27,29–32], smoking cessation (n = 1), [26,28], and a combination of smoking cessation and medication adherence (n = 1) [28]. No qualitative studies investigated the experiences of people from minority ethnic communities with long-term oxygen therapy and vaccinations. Further study characteristics are detailed in Table 1.

**Table 1:** Characteristics of the included studies.

Author, Year	Country	Method of data collection	Type of intervention	Sample size/Age (years)/Sex	Minority ethnic groups	Aim	Main themes extracted from the original study
Poureslami et al, 2015[26]	Canada	Semi-structured interviews	Smoking cessation	91 participants Median age: 75 years Male: 71	Chinese people living in Canada	To identify and differentiate the smoking habits, beliefs, perceptions and attempts to give up smoking between those who had achieved this and those who continued to smoke.	• Reasons for smoking initiation • Previous quitting attempts and reasons • Advantages and disadvantages of smoking • Smoking-related health beliefs
Levack *et al* (2016)[32]	New Zealand	Semi-structured focus group and individual interviews	Pulmonary rehabilitation	25 participants Age range: 40–79 years Male: 13	Māori: 15 non-Māori: 10	Examine factors influencing uptake of pulmonary rehabilitation by Māori with COPD in New Zealand.	• Past experiences. • attitudes and expectations • access issues. • program experiences. •
Glasser *et al* (2016)[28]	USA	Focus groups interviews	Smoking cessation, Medication adherence	25 participants Age range: 41–89 years Male: 8	Hispanic: 18 African American: 3 White: 4	Understand the COPD patient viewpoint in terms of experiences with care from the Clinic and to offer suggestions for future patient care.	• Problems living with COPD. • Coping with complexities of comorbid illnesses. • Challenges of quitting smoking and maintaining cessation. • Dealing with second-hand smoke. • Beliefs about quitting smoking. • Difficulty paying for and obtaining medications. • Positive experiences regarding medications. • Difficulties using sleep machines at home. • Expressions of disappointment with the departure of their doctors. • Overall satisfaction with the clinic providers
Brighton *et al* (2020)[30]	UK	Semi-structured interviews	Pulmonary rehabilitation	19 participants Age range: 58–88 years Male: 9	Asian, Black, or Mixed: 3 White British or Irish: 16	Explore motivation for, and barriers to, continued participation in pulmonary rehabilitation.	• Striving to adapt to multidimensional loss. • Tensions of balancing support with independence • Pulmonary rehabilitation is a challenge worth facing. • Overcoming unpredictable disruptions to participation
Early et al, 2020[31]	UK	Semi-structured interviews and focus groups	Pulmonary rehabilitation	42 participants Male: 23 Age: not reported by study authors	White British: 35 South Asian: 7	To generate a theory-informed understanding of enablers and barriers to PR referral and uptake from primary care.	Not formally reported but it included discussion of barriers and enablers to pulmonary rehabilitation including: Language, family, and gender. SA contacted the study authors asking for the qualitative data and the provided South Asians’ original quotes
Pekmezaris et al, 2020[29]	USA	Focus groups	Pulmonary rehabilitation	20 CAB members Characteristics not reported by study authors	Hispanic and African American	To explore the perspectives of community stakeholders through the conduct of 2 focus groups of members of a CAB established to ensure patient centeredness at every phase of a mixed methods, comparative effectiveness research study of in-home tele-pulmonary rehabilitation.	Equipment Changes • Recruitment Changes • Study Logistics • Self-Efficacy • Access
Polo *et al*, 2023[27]	USA	Thirty-nine structured interviews, plus two focus group with 10 participants	Pulmonary rehabilitation	39 participants Mean age: 64.10 years Male: 12	Hispanic: 11 African American: 28	Understanding of (1) the barriers to initiating PR despite a referral to PR and (2) the barriers to participating in >1 PR session once started.	• Approach, Recruitment and Enrolment • Experiences Unique to Those Participants Randomized to SPR. • Experiences Unique to Those Participants Randomized to Tele-PR. • Experiences that are Relevant to either Tele-PR or SPR. • Post Intervention PR Maintenance

Key: USA = United States of America; UK = United Kingdom; PR = pulmonary rehabilitation; CAB = Community advisory board; COPD = Chronic obstructive pulmonary disease; Tele-PR = Telehealth‐delivered pulmonary rehabilitation; SPR = standard pulmonary rehabilitation

### Study quality

Quality assessment for the included studies was conducted by (SA and AR-B) using the Critical Appraisal Skills Programme (CASP) tool for qualitative research [23]. All the included articles met the criteria for clarity of research aims, consideration of ethical issues, data collection, and reporting of study results [26–32]. However, four of the studies lacked sufficient details regarding the relationship between the researcher and the participants [26,27,29,31]. Of the included studies, Brighton *et al*. [30], Levack *et al*. [32], and Glasser *et al*. [28] were found to have the highest quality. Details of the quality appraisal in Table 2.

**Table 2:** Quality appraisal.

Author & Year	Critical Appraisal Skills Tool Screening Questions										Comments
	1. Was there a clear statement of the aims of the research?	2. Is qualitative methodology appropriate?	3. Was the research design appropriate to address the aims of the research?	4. Was the recruitment strategy appropriate to the aims of the research?	5. Was the data collected in a way that addressed the research issue?	6. Has the relationship between researcher and participants been adequately considered?	7. Have ethical issues been taken into consideration?	8. Was the data analysis sufficiently rigorous?	9. Is there a clear statement of findings?	10. How valuable is the research?	
	*Yes/No/Can’t tell*										
Brighton *et al* (2020)[30]	Yes	Yes	Yes	Yes	Yes	Yes	Yes	Yes	Yes	Yes	-
Levack *et al* (2016)[32]	Yes	Yes	Yes	Yes	Yes	Yes	Yes	Yes	Yes	Yes	
Glasser *et al* (2016)[28]	Yes	Yes	Yes	Yes	Yes	Yes	Yes	Yes	Yes	Yes	
Polo *et al* (2023)[27]	Yes	Yes	Yes	Yes	Yes	Can’t Tell	Yes	Yes	Yes	Yes	Q4, Q5, Q6, Q8, Q9: Reported in the supplementary file of the study.
Pekmezaris et al, (2020)[29]	Yes	Yes	Yes	Can’t Tell	Yes	Can’t Tell	Yes	Yes	Yes	Yes	
Poureslami et al, (2015)[26]	Yes	Yes	Yes	Can’t Tell	Yes	No	Yes	Can’t Tell	Yes	Yes	Q5, Data saturation not reported.
Early et al, (2020)[31]	Yes	Yes	Yes	Yes	Yes	No	Yes	Can’t Tell	Yes	Yes	

### Findings

**Fig 2:**
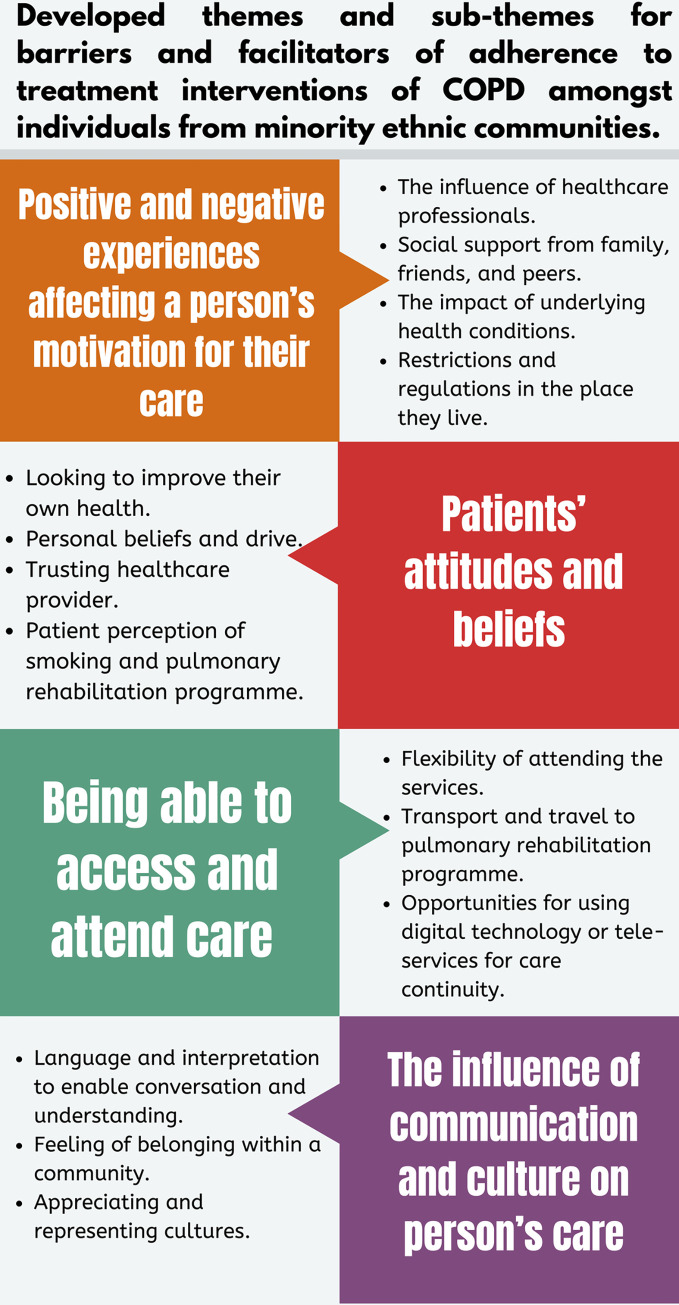
Developed themes and sub-themes for barriers and facilitators of adherence to treatment interventions of COPD amongst individuals from minority ethnic communities.

Four overarching themes and 14 sub-themes were developed in this qualitative evidence synthesis: 1) positive and negative experiences affecting a person’s motivation for their care, 2) patient attitude and beliefs, 3) being able to access and attend care, and 4) the influence of communication and culture on a person’s care. These themes and sub-themes appear key in establishing a deeper understanding of barriers and facilitators of adherence to treatment interventions amongst people living with COPD from minority ethnic communities (Fig 2). A detailed breakdown of each theme can be viewed in the supporting information (S4 Table).

### Theme 1: Positive and negative experiences affecting a person’s motivation for their care

*Subtheme 1*: *The influence of healthcare professionals*. Healthcare providers were seen to play a vital role in supporting and motivating individuals from minority ethnic communities living with COPD across five articles [26–28,31,32]. Healthcare providers could positively and/or negatively influence a person to attend pulmonary rehabilitation programmes or quit smoking [27,30,32]. Further, a female participant of Hispanic ethnicity recounted a positive experience with her nurse in acquiring and adhering to COPD medication [28].

*“Was there anything about the class that made you nervous before you started, or when you first started going? The exercise. I used to think “oh,” you know. and the physio will say “no, keep going, keep going,” you know, you think “I can’t breathe.” I used to be afraid of the breathing.”(Māori) [32].*

*“I told the doctor that I don’t have the strength and the will power (to quit smoking) and he said*: “*I cannot deal with you*. *If you don’t quit smoking, I will see you with an oxygen tank in one year.” And that made me feel very bad.” (Hispanic) [28].*

Many participants of South Asian, Hispanic, and African American ethnicities reported that they had never been referred to pulmonary rehabilitation or given the opportunity to discuss attending the programme [27,31]. The lack of continuity in healthcare provider-patient relationships and insufficient consultation time caused people living with COPD to miss out on pulmonary rehabilitation, a valuable opportunity to improve their symptoms, and resulted in delays in accessing crucial interventions.

*“No, I have never been referred. They just ask me to continue doing some exercises. I get a lot of exercises since I do all the housework. But I have never been told what you are saying.” (British Pakistani)[31].*

“*Like I said*, *my doctor never told me anything about that [PR]*. *I mean*, *he could tell his patients when they have severe–chronic breathing*, *respiratory infections*, *and things like that*. *He should advise his patients to just give it a try*. *If he would have told me to go*, *I would have went*.*” (Hispanic)* [27].

On the other hand, some participants reported positive experiences with healthcare providers that enabled them to engage in pulmonary rehabilitation and adherence to medication [28,31].

*“What I liked and enjoyed most was that it gave me a routine and took me out of the house, I felt good mingling with other people, I enjoyed interacting with them, it helped me relax.” (British Pakistani) [31].*

*“I don’t have problems (with medications) because I have nurses who come to my house*. *They come every week on Tuesdays and Fridays*. *They are lovely*, *lovely*, *responsible people*.” *(Hispanic)* [28].

*Subtheme 2*: *Social support from family*, *friends*, *and peers*. Social networks can also play a role in motivating uptake and adherence to pulmonary rehabilitation and smoking cessation amongst individuals from minority ethnic communities diagnosed with COPD [26,28–30,32]. Participants from Chinese ethnicity in the study by Poureslami *et al*. conducted in Canada cited the detrimental effect of family and friends as justification for their decision to start smoking or their struggle to quit [26]. People of Māori ethnicity indicated the importance of a culturally familiar environment and being with peers who had similar cultural perspectives in their tendency to attend pulmonary rehabilitation programmes [32].

*“I quit once, then I started to smoke again because smoking helps me build close relationships with my friends when we play cards” (Chinese in Canada)[26].*

“After coming to Canada, I was forced to quit smoking by my family, my daughters told me to quit and yelled at me.” (Chinese in Canada)[26].

*“How it was for me?–I felt very left out, I felt like–being a Māori, you know. They were all in their own individual group, you know, the Pākehā [non-Māori New Zealanders]… I stuck out like a sore thumb, being the only Māori there.” (Māori) [32].*

*Subtheme 3*: *The impact of underlying health conditions*. Participants of Māori ethnicity in the study by Levack *et al*. reported the severity of COPD as a barrier to attending and completing the pulmonary rehabilitation programme due to severe symptoms limiting their mobility [32]. Additionally, Poureslami *et al*. *and* Glasser *et al*. found that worsening symptoms of COPD motivated people from minority ethnic communities to quit smoking, as it helped them better manage the disease and improve their quality of life [26,28].

*“And that exercise in the hospital gym was rubbish. We had to be there at nine o’clock in the morning, I mean anyone with COPD or severe emphysema know, that’s not a really for us. It takes me over an hour just to have a shower.” (Māori) [32]*

“I cut down smoking because I hospitalised for my lung problem, then I cut my one-pack a day smoking [sic] to three packs a month.” (Chinese in Canada)[26].

*“I know bad health well. I was in intensive care and I believed that I was going to die. When you see this you quit cigarettes. You say, “Oh my God, if you continue this way you are going to die.” (Hispanic) [28].*

Despite that, the beneficial impact of smoking cessation and pulmonary rehabilitation on the health of individuals living with COPD has been identified as a facilitating factor for adherence amongst people of Māori ethnicity, and Chinese people who are resident in Canada [26,32].

*“[To start with] I just thought “oh, what we’re going–going to get out of it?” But I thought I’ll keep going. But then I said to them, when I’d been to it for a few times, I used to say, “oh, I must admit, it has been doing me good.” I said–before I used to think oh exercise was just a load of–it’s not going to help you, you know, with all the breathlessness and all that. It’s not going to help you.” (Māori) [32].*

“When you quit smoking, your lung function improves, my doctor said.” (Chinese in *Canada) [26].*

*Subtheme 4*: *Restrictions and regulations in the place they live*. Governmental regulation on smoking, including taxes and the prohibition of smoking in public areas, helped individuals of Chinese ethnicity diagnosed with COPD decide to quit smoking after immigrating to Canada [26].

*“I quit smoking after coming to Canada and observed that it’s not culturally good habit to smoke.” (Chinese in Canada) [26].*

*“Smoking is expensive.” (Chinese in Canada) [26].*

### Theme 2: Patients’ attitudes and beliefs

*Subtheme 1*: *Looking to improve their own health*. People’s perspectives about the benefits and health improvements resulting from participating in pulmonary rehabilitation programmes were demonstrated as a facilitator for adherence by some participants from minority ethnic communities; namely, Asian, African American, and Hispanic ethnicities [29,30].

*“I’ll go and try anything, I’ve done that a lot, I thought I’ll get there somehow but do something positive. As long as I’m doing something positive to help myself, if you like, I’ll do it.” (Asian, Black, or Mixed) [30].*

*“I liked them a lot, I wanted to see if this worked for me. It wasn’t easy at first because I wasn’t used to it, but now I can even do them on my own.” (Hispanic or African American) [29].*

*Subtheme 2*: *Personal beliefs and drive*. Numerous participants with Māori ethnicity pointed out the impact of their beliefs and expectations on their decision-making regarding their participation in and continuation of pulmonary rehabilitation programmes [32]. Levack *et al*. stated that negative personal perceptions were attributed to attitudes that drives expectations and thoughts about the programmes [32].

*“I used to have a bad attitude. I used to think, oh, exercise ain’t going to help me, you know… At first I really had a bit of a negative attitude to it. Cause I have negative attitude to everything. and I’m like “nah, I don’t want to go to that.” You know, I lose interest in things quick. real quick.” (Māori) [32].*

*Subtheme 3*: *Trusting healthcare provider*. South Asian participants in the study by Early *et al*. exhibit a strong inclination towards engaging and completing pulmonary rehabilitation programmes by placing significant value on advice provided by healthcare professionals [31]. The authors described trusting and following healthcare recommendations as an enabler to pulmonary rehabilitation uptake [31].

*“No they did not tell me anything at all [about what happens at PR]. They said it’s a very good thing, do go. They said will you go if we refer? I said yes.” (British Pakistani) [31]*

*“Yes I will be interested. I will definitely go if they ask me to.” (British Pakistani) [31]*

*Subtheme 4*: *Patient perception of smoking and pulmonary rehabilitation programme*. People of Chinese ethnicity who smoke and live with COPD in Canada discussed various reasons to continue smoking in the study by Poureslami *et al* [26]. Many participants hold the misconception that smoking can alleviate stress and anxiety and fight viruses [26]. They also thought that smoking benefits outweighed its risks and it is considered an effective way for re-energisation and alertness [26]. Additionally, a lack of belief in the advantages of pulmonary rehabilitation was reported as a barrier to attending the programme amongst participants of British Pakistani ethnicity [31].

*“Smoking helps with disinfection” (Chinese in Canada) [26].*

‘‘I felt more relaxed when I smoked” (Chinese in Canada) [26].

“Honestly, I don’t think there’s anything bad with smoking–but my doctor told me to stop” (Chinese in Canada) [26].

*“The exercises were not directly related to my lungs, they showed me exercises which was generally good for my body, there were all kinds of exercises and they taught me bits of all types of exercises. I would say they were excellent; the exercises are somewhat helpful. I have been asked to do them at home, I try to do them regularly but I am unable to do all the exercises the way they showed us in the classes.” (British Pakistani) [31].*

### Theme 3: Being able to access and attend care

*Subtheme 1*: *Flexibility of attending the services*. The flexibility of the programmes impacted the adherence and engagement to the services amongst people from minority ethnic communities. People of Māori, and Asian, ethnicities living with COPD may find their motivation eroded by the timing and duration of pulmonary rehabilitation programmes because of many conflicting obligations, such as those to religion, family and work [30,32].

*“I did Wednesday and Friday. But then I couldn’t cope with Friday.”. “I did go, and I said, ‘I can’t do Fridays.” (Asian, Black, or Mixed)[30].*

*“I found that early morning was not good for me*. *I couldn’t concentrate on what they were doing*. *and the last thing I wanted to do was walk*.*” (Māori)* [32].

*Subtheme 2*: *Transport and travel to pulmonary rehabilitation programme*. Participants from Māori and South Asian ethnic communities highlighted transportation issues related to attending pulmonary rehabilitation programmes, including cost, distance, and reliance on family [31,32]. Lacked personal transport due to financial constraints and limited access to public transport influencing uptake of pulmonary rehabilitation by people of Māori ethnicity [32]. Similarly, individuals from South Asian ethnic communities identified that limited capital resources and dependence on relatives for transportation as barriers to accessing these services [31].

*“I said to her “am I the only Māori coming?” And she said “well there’s meant to be others, but they’re not coming.” And I said “probably because you have to find your own way.” (Māori) [32].*

*“That I can only be sure after I go, but I will try to complete the course. I was unsure who will take me there. But my daughter said she will. In my locality its once a week only.” (British Pakistani) [31].*

*Subtheme 3*: *Opportunities for using digital technology or tele-services for care continuity*. Pekmezaris *et al*. and Polo *et al*. investigated the acceptability and usability of telehealth pulmonary rehabilitation among people diagnosed with COPD from Hispanic and African American ethnic communities [27,29]. Being able to access pulmonary rehabilitation services from home using tablets facilitated adherence and enabled people of minority ethnic communities to overcome certain attendance-related obstacles such as language [29]. Tablets were modified to use icons, making it easier for patients to access various aspects of the app through point-and-click interaction, regardless of their primary language [29]. However, some participants expressed their feelings of being abandoned because they perceived that the programme had been abruptly withdrawn from their access at the end of the study [27].

*“You don’t have to struggle… with all the equipment, everything is set up just right for the patient to access everything.” (Hispanic or African American) [29].*

*“They give the equipment in order to help you, and then they take it back from you. That breaks the morale of the whole program, you know. It’s like building you up and breaking you down” (African American) [27].*

### Theme 4: The influence of communication and culture on a person’s care

*Subtheme 1*: *Language and interpretation to enable conversation and understanding*. Language barrier was found to have a negative impact on how individuals from minority ethnic communities receive and understand information about pulmonary rehabilitation programmes. This was particularly noted among people from South Asian ethnic communities who mostly required interpreters during appointments due to their limited language proficiency [31]. Reliance on interpreters presented challenges for healthcare providers to accurately interpret emotional cues and fully understand the conversation [31]. Consequently, ineffective coordination of interpretation services resulted in patients feeling marginalised or misunderstood, leading to influence their uptake and adherence to pulmonary rehabilitation.

*“Even I did not know much about it till you explained. It is also possible that they did explain but I did not get it properly. They were in English.” (British Pakistani) [31]*

“I could not understand [the education part] them. But I had my daughter with me she would explain things to me. I could not understand English.” (British Pakistani) [31].

*“We are not very fluent in English. It is possible that this is why people do not go. I don’t understand 100%, but even I were to understand only 50% it is still beneficial. There is no harm.” (British Pakistani) [31].*

*Subtheme 2*: *Feelings of belonging within a community*. People of Māori ethnicity living with COPD placed considerable value on Whakawhanaungatanga, which is the Māori approach of building and maintaining relationships [32]. Individuals from Māori ethnic community emphasised the importance of connecting with peers who shared similar cultural perspectives, as this influenced their decision to uptake pulmonary rehabilitation [32].

*“And it just that whanaungatanga [connecting with others] time is very important, how everyone feels. It’s something personal to yourself. But some get to say more than others… But it’s a kind of down to our level, and it’s good to bringing the tikanga aspect side of things, tikanga Māori, our waiata, how we do things, who with, and in a place that we feel good in being.” (Māori) [32].*

*Subtheme 3*: *Appreciating and representing cultures*. Individuals from minority ethnic communities have demonstrated an intense appreciation for their cultural heritage, which may have an impact on their engagement in pulmonary rehabilitation programmes. For instance, People from South Asian communities living with COPD cited the presence of mixed-sex classes as a barrier to attending pulmonary rehabilitation programmes [31].

*“I did not like this [mixed classes]. Men should be separate from women. This was the most difficult part.” (British Pakistani) [31].*

## Discussion

To our knowledge, this is the first meta-ethnographic systematic review exploring barriers and facilitators of adherence to treatment interventions of COPD amongst individuals from minority ethnic communities. Four overarching themes (third-order constructs) were developed in this meta-ethnography, which appreciated the barriers and facilitators of adherence to treatment interventions for people living with COPD from ethnic minority communities: 1) Positive and negative experiences affecting a person’s motivation for their care, 2) Patient attitude and beliefs, 3) Being able to access and attend care, and 4) The influence of communication and culture on a person’s care.

Patients’ experiences with healthcare professionals, family and friends, underlying health conditions, and the governmental regulations in the place they live were reported to have both negative and positive influences on adherence to treatment interventions of COPD amongst people from minority ethnic communities. People from Māori and South Asian ethnicities living with COPD emphasised the pivotal role of the support they received from their healthcare providers and social networks in facilitating their participation in pulmonary rehabilitation programmes [31,32]. Several studies that did not report the ethnicity of participants have also highlighted the significance of healthcare professionals, peer groups, and family support in enhancing engagement in pulmonary rehabilitation programmes [33–36]. The beneficial roles of healthcare providers, family, and peers were also seen to motivate individuals living with COPD to quit smoking, mainly among people from African American, and Hispanic ethnicities and people of Chinese ethnicity living in Canada [26,28]. Consistently, Twyman *et al*. reported the absence of support as a barrier to smoking cessation amongst individuals from vulnerable groups [37]. The term “vulnerable groups” is defined by Twyman *et al*. as ‘*groups that are more likely to experience social and material disadvantage due to lower income*, *cultural differences and social exclusion’* [37].

Patients’ attitudes and beliefs were also identified to be both barriers and facilitators to adherence to treatment interventions of COPD amongst minority communities in the following subthemes: 1) Looking to improve their own health, 2) Personal beliefs and drive, 3) Placing trust in healthcare providers, 4) Patients’ perception of smoking and pulmonary rehabilitation programme. People from Asian, Black, Hispanic, and African American communities were motivated to participate in pulmonary rehabilitation programmes to improve their health, and consequently, their quality of life [29,30]. These findings echo those of previous reviews, which reported that people with COPD, irrespective of their ethnicity, were motivated to participate in pulmonary rehabilitation because they found it an opportunity for health transition and control of their functional status [35,37,38]. Placing trust in the advice provided by healthcare professionals was found to be a facilitator for attending pulmonary rehabilitation programmes, particularly evident among people from South Asian community [31]. These results further support the ideas shared by Sohanpal *et al*., who indicated that establishing trust between patients and healthcare providers is associated with a heightened attendance rate to pulmonary rehabilitation programmes amongst individuals living with COPD [38]. However, it is worth noting that the findings by Sohanpal *et al*. were not specifically categorised by ethnicity.

This review also indicated that patients’ perception of COPD treatment interventions impacted their adherence. Participants from Chinese ethnicity diagnosed with COPD reported using smoking for stress and anxiety management [26]. Further, members of this community also shared beliefs of getting worse disease symptoms following smoking cessation [26]. These results are consistent with Patterson *et al*., who reported the fear of discomfort or deteriorating wellness, and a low awareness level about the risk of smoking, as barriers to quitting in a sample of people of African-American ethnicity living with COPD [39]. This meta-ethnography also demonstrated lower enrolment rates for pulmonary rehabilitation programmes amongst individuals from South Asian communities, because they did not find it enjoyable [31]. The impact of enjoyment on adherence to pulmonary rehabilitation programmes has been previously been identified in wider qualitative studies, although without reporting the participants’ ethnicity [33,36]. Future research may seek to further explore these barriers around enjoyment, particularly whether culturally-competent design and tailoring could be implemented to better suit the needs of people from different ethnicities [40,41].

Difficulties associated with access to pulmonary rehabilitation, such as timing and duration of the programme, geographical distance to the programme, and transportation, were reported in this review by participants from Asian, Black, and Māori ethnic communities [30–32]. However, several studies reported accessibility as a major barrier to pulmonary rehabilitation amongst individuals living with COPD, despite their ethnicity [15,35,38,42–46]. Levack *et al*. reported the association between minority ethnic communities and possible lower socioeconomic status, making individuals more susceptible to transportation issues [32]. Moreover, Early *et al*. suggested the urgent need for further research to understand patients’ experiences with access to pulmonary rehabilitation particularly in minority ethnic communities because most of the qualitative studies do not report the ethnicity of participants [42]. Emerging digital advantages were noted in this work, particularly the use of telehealth to deliver pulmonary rehabilitation programmes, which was supported by people from Hispanic and African-American communities [27,29]. Further research could seek to explore other digital delivery methods, noting participant preferences during- and at completion- of such programmes [27]. This was also noted in a previous systematic review with the standard pulmonary rehabilitation programme, the patients defined the end of the programme as ‘the end of any formal support’ and ‘temporal security and support’ [35].

Language was identified by this review as a barrier hindering communication between healthcare providers and people with COPD from minority ethnic communities. Insufficient verbal concordance made it difficult for patients to understand the provided consultation and discouraged them from asking further questions. Several studies reported the impact of language barriers and low health literacy on patients’ non-adherence and poor self-management of airway diseases such as asthma and COPD [47–49]. However, Poureslami *et al*., emphasise that individuals from minority ethnic communities are disproportionately impacted by the disadvantages of limited health literacy, language, and cultural barriers [47]. These results are consistent with those of Auyeung *et al*., who suggested that language and cultural barriers influenced adherence to pulmonary rehabilitation programmes by people of Chinese ethnicity living with COPD in Sydney [43]. The majority of qualitative studies investigated the barriers to adherence to pulmonary rehabilitation programme are excluded patients who are unable to communicate in English [46]. Thus, suggesting that little is known about the influence of language and cultural barriers to enrol in pulmonary rehabilitation programmes. These findings underscore the need for further exploration of the impact of language and cultural barriers on adherence to treatment interventions for COPD among individuals from minority ethnic communities. It also highlights the critical need for designing and implementing targeted interventions that address these specific vulnerabilities within minority ethnic communities.

### Strengths and limitations

To the best of our knowledge, this meta-ethnographic systematic review is the first to explore the barriers and facilitators affecting adherence to COPD treatment interventions among individuals from minority ethnic communities. Secondly, eMERGe guidelines were followed in reporting the findings of this review. Furthermore, authors (SA, AR-B, HN, AH, and MC) were independently screened studies using pre-designed inclusion criteria. SA and AR-B independently appraised the quality of the studies, and participated in the phases of translation, synthesising translation, and expressing the synthesis, and came to a consensus through discussion.

This review was restricted to studies published in English only; as such, the authors recognise this may have resulted in excluding findings from potentially relevant publications written in other languages. Despite the broad inclusion criteria used in this review, a limited number of qualitative studies were retrieved that reported qualitative data in accordance to participant ethnicity (i.e. matching quotes to participant demographics). As such, the authors make suggestion for more transparent qualitative data reporting. Included studies were only exploring patients’ experience with pulmonary rehabilitation and smoking cessation. Lastly, all minority ethnic communities were treated as a single entity, with any differences and complexities between various ethnic communities being ignored. To further understand the variation of experiences within each minority ethnic community, it would be valuable to identify barriers and facilitators of adherence to treatment interventions of COPD in each minority ethnic community in future studies.

### Points for clinical practice, and questions for future research

The implications of the findings of this review have significant relevance for both clinical practice and future research. In practice, understanding the barriers and facilitators experienced by people who are from minority ethnic communities with treatment interventions of COPD allows healthcare providers to tailor interventions and support mechanisms to better address the variety of needs of these communities. By recognising and addressing cultural, social, and linguistic factors that impact decisions about COPD treatment, healthcare providers could enhance patient adherence and eventually improve treatment outcomes. Moreover, this review highlights the importance of developing rapport and trust between healthcare professionals and individuals living with COPD who are from minority ethnic communities, emphasising the need for ethnic-sensitive care delivery. For future research, these findings indicated the necessity of further exploration into the unique challenges faced by people from minority ethnic communities in managing COPD. It is crucial to develop patient-centred interventions that address any disparities while considering the influence of language and culture. Previous qualitative studies have often excluded non-English speakers, leaving a significant gap in understanding how these factors impact the management of COPD amongst people of minority ethnic communities. Therefore, qualitative research that involves non-English speakers of minority ethnic communities is essential for a comprehensive understanding of the influence of ethnicity on adherence to treatment interventions for COPD.

## Conclusion

This meta-ethnography systematic review has developed four main themes of barriers and facilitators of adherence to treatment interventions of COPD among people from minority ethnic communities. The findings of this review demonstrate the need for more investigation into the influence of language and cultural characteristics on adherence to treatment interventions for COPD. This deeper understanding could support the creation of tailored protocols and strategies to enhance treatment adherence and improve disease outcomes amongst people living with COPD who are from minority ethnic communities. By addressing these factors, healthcare professionals could also develop more effective, culturally sensitive interventions that meet the diverse needs of individuals from minority ethnic communities.

## Supporting information

S1 Table The eMERGe checklist for meta-ethnography. (DOCX)

S2 Table Search strategy. (DOCX)

S3 Table A numbered table of all 639 studies identified in the literature search. (DOCX)

S4 Table Result of the qualitative data synthesis. (DOCX)
