# Correction: Barriers and facilitators of adherence to treatment interventions for COPD amongst individuals from minority ethnic communities: Meta-ethnography

**DOI:** 10.1371/journal.pone.0334058

**Published:** 2025-10-08

**Authors:** Sarah Alamer, Anna Robinson-Barella, Matthew Cooper, Hamde Nazar, Andy Husband

The second author’s name is spelled incorrectly. The correct name is: Anna Robinson-Barella. The correct citation is: Alamer S, Robinson-Barella A, Cooper M, Nazar H, Husband A (2025) Barriers and facilitators of adherence to treatment interventions for COPD amongst individuals from minority ethnic communities: Meta-ethnography. PLoS ONE 20(2): e0318709. https://doi.org/10.1371/journal.pone.0318709

## References

[1] Alamer S, Robinson-Barellla A, Cooper M, Nazar H, Husband A. Barriers and facilitators of adherence to treatment interventions for COPD amongst individuals from minority ethnic communities: Meta-ethnography. PLoS One. 2025;20(2):e0318709. doi: 10.1371/journal.pone.0318709 39928635 PMC11809908

