# Supplementary material for: Barriers and facilitators of adherence to treatment interventions for COPD amongst individuals from minority ethnic communities: Meta-ethnography
Source: PLoS One. 2025 Feb 10;20(2):e0318709. doi: 10.1371/journal.pone.0318709 (PMC11809908; doi:10.1371/journal.pone.0318709)
Supplement: S1 Table — (DOCX) [file pone.0318709.s001.docx]

**S1 Table: The eMERGe checklist for meta-ethnography**

| **eMERGe** **criteria** | | **Pages** |
| --- | --- | --- |
| **Phase 1: Selecting meta-ethnography and getting started** | 1. **Rationale and context for the meta-ethnography**   Describe the gap in research or knowledge to be filled by the meta-ethnography, and the wider context of the meta-ethnography | 5-6 |
|  | 1. **Aim(s) of the meta-ethnography**   Describe the meta-ethnography aim(s) | 6 |
|  | 1. **Focus of the meta-ethnography**   Describe the meta-ethnography question(s) (or objectives) | 6 |
|  | 1. **Rationale for using meta-ethnography**   Explain why meta-ethnography was considered the most appropriate qualitative synthesis methodology | 6 |
| **Phase 2: Deciding what is relevant** | 1. **Search strategy**   Describe the rationale for the literature search strategy | 7, (Supplementary S2) |
|  | 1. **Search processes**   Describe how the literature searching was carried out and by whom |  |
|  | 1. **Selecting primary studies**   Describe the process of study screening and selection, and who was involved | 7-8 |
|  | 1. **Outcome of study selection**   Describe the results of study searches and screening | 10, Figure 1 |
| **Phase 3: Reading included studies** | 1. **Reading and data extraction approach**   Describe the reading and data extraction method and processes | 9 |
|  | 1. **Presenting characteristics of included studies**   Describe characteristics of the included studies | 10-13, Table 1 |
| **Phase 4: Determining how studies are related** | 1. **Process for determining how studies are related**   Describe the methods and processes for determining how the included studies are related: Which aspects of studies were compared AND How the studies were compared | 9,10 |
|  | 1. **Outcome of relating studies**   Decribe how studies relate to each other | 9 |
| **Phase 5: Translating studies into one another** | 1. **Process of translating studies**   Describe steps taken to preserve the context and meaning of the relationships between concepts within and across studies  Describe how the reciprocal and refutational translations were conducted  Describe how potential alternative interpretations or explanations were considered in the translations | 9,10 |
|  | 1. **Outcome of translation**   Describe the interpretive findings of the translation | 9,10 |
| **Phase 6: Synthesising translations** | 1. **Synthesis process**   Describe the methods used to develop overarching concepts (‘synthesised translations’). Describe how potential alternative interpretations or explanations were considered in the synthesis | 10 |
|  | 1. **Outcome of synthesis process**   Describe the new theory, conceptual framework, model, configuration or interpretation of data developed from the synthesis | 15-24 |
| **Phase 7: Expressing the synthesis** | 1. **Summary of findings**   Summarise the main interpretive findings of the translation and synthesis and compare them to existing literature | 24-27 |
|  | 1. **Strengths and limitations**   Reflect on and describe the strengths and limitations of the synthesis:  For example, describe how the synthesis findings were influenced by the nature of the included studies and how the meta-ethnography was conducted | 27-28 |
|  | 1. **Recommendation and conclusion**   Describe the implications of the synthesis | 28-29 |
