# Supplementary material for: Barriers and facilitators of adherence to treatment interventions for COPD amongst individuals from minority ethnic communities: Meta-ethnography
Source: PLoS One. 2025 Feb 10;20(2):e0318709. doi: 10.1371/journal.pone.0318709 (PMC11809908; doi:10.1371/journal.pone.0318709)
Supplement: S2 Table — (DOCX) [file pone.0318709.s002.docx]

**S2 Table: Search strategy**

**CINAHL**

S9 S1 AND S7 AND S8

S8 S2 OR S3 OR S4 OR S5 OR S6

S7 Barriers OR facilitators OR enablers OR “influence factors”

S6 “Medication adherence” OR “Medication compliance” OR “medication non-adherence”

S5 “Long term oxygen therapy” OR “LTOT”

S4 “Influenza vaccine” OR “Influenza vaccination” OR “Flu vaccine”

S3 Pulmonary rehabilitation

S2 “Smoking cessation” OR “Quit smoking”

S1 “COPD” OR “Chronic obstructive pulmonary disease” OR “Chronic lung disease”

**PsycINFO**

**30** 22 and 29

**29** 23 or 24 or 25 or 26 or 27 or 28

**28** MIXED STUDY.mp.

**27** exp Focus Group/ or FOCUS GROUP.mp.

**26** exp Interviews/ or TELEPHONE INTERVIEW.mp.

**25** exp Semi-Structured Interview/ or SEMI STRUCTURED INTERVIEW.mp.

**24** QUALITATIVE RESEARCH.mp. or exp Qualitative Methods/

**23** QUALITATIVE ANALYSIS.mp.

**22** 14 and 21

**21** 15 or 16 or 17 or 18 or 19 or 20

**20** ENABLER*.mp.

**19** DETERMINANT* mp.

**18** CHALLENGES.mp.

**17** INFLUENCING FACTOR*mp.

**16** FACILITATOR*.mp.

**15** BARRIER* mo.

**14** 4 and 13

**13** 5 or 6 or 7 or 8 or 9 or 10 or 11 or 12

**12** MEDICATION NONADHERENCE.mp.

**11** exp Treatment Compliance/ or MEDICATION ADHERENCE.mp.

**10** FLU VACCINE.mp.

**9** INFLUENZA VACCIN* mp.

**8** LTOT.mp.

**7** long term oxygen therapy.mp.

**6** Smoking cessation.mp. or exp Smoking Cessation/

**5** Pulmonary rehabilitation.mp.

**4** 1 or 2 or 3

**3** COPD.mp.

**2** CHRONIC OBSTRUCTIVE LUNG DISEASE.mp.

**1** CHRONIC OBSTRUCTIVE PULMONARY DISEASE.mp. or exp Chronic Obstructive Pulmonary Disease/

**Medline**

**45** 33 and 44

**44** 34 or 35 or 36 or 37 or 38 or 39 or 40 or 41 or 42 or 43

**43** Mixed method$.mp.

**42** Qualitative study.mp.

**41** Thematic analysis.mp.

**40** Mixed method study.mp.

**39** Focus group.mp.

**38** Interview.mp.

**37** Telephone interview.mp.

**36** Semi structured interview.mp.

**35** Qualitative research.mp. or exp Qualitative Research/

**34** Qualitative analysis.mp.

**33** 20 and 32

**32** 21 or 22 or 23 or 24 or 25 or 26 or 27 or 28 or 29 or 30 or 31

**31** Engagement.mp.

**30** Uptake.mp.

**29** Referral.mp.

**28** Accessibility.mp.

**27** Feasibility.mp.

**26** Challenge$.mp.

**25** Adherence factor$.mp.

**24** Determinant$.mp.

**23** Influencing factor$.mp.

**22** Facilitator$.mp.

**21** Barrier$.mp.

**20** 6 and 19

**19** 7 or 8 or 9 or 10 or 11 or 12 or 13 or 14 or 15 or 16 or 17 or 18

**18** Vaccination.mp. or Vaccination/

**17** Influenza vaccination.mp.

**16** Influenza vaccine.mp. or Influenza Vaccines/

**15** Smoking cessation agent$.mp. or exp Smoking Cessation Agents/

**14** Quitting smoking.mp.

**13** Smoking cessation.mp. or exp Smoking Cessation/

**12** Pulmonary rehabilitation.mp.

**11** Oxygen Inhalation Therapy/ or LTOT.mp.

**10** long term oxygen therapy.mp.

**9** Inhaler$ adherence.mp.

**8** exp "Treatment Adherence and Compliance"/

**7** Medication adherence.mp. or exp Medication Adherence/

**6** 1 or 2 or 3 or 4 or 5

**5** chronic obstructive lung disease$.mp.

**4** chronic obstructive pulmonary disease$.mp.

**3** Chronic obstructive lung disease.mp.

**2** COPD.mp.

**1** Chronic obstructive pulmonary disease.mp. or exp Pulmonary Disease, Chronic Obstructive/

**EMBASE**

**44** 32 and 43

**43** 33 or 34 or 35 or 36 or 37 or 38 or 39 or 40 or 41 or 42

**42** Mixed method$.mp.

**41** Thematic analysis.mp. or exp thematic analysis/

**40** Mixed method study.mp.

**39** Focus group.mp.

**38** exp interview/ or interview.mp.

**37** Telephone interview.mp. or exp telephone interview/

**36** Semi structured interview.mp. or exp semi structured interview/

**35** Qualitative study.mp.

**34** exp qualitative research/ or qualitative research.mp.

**33** Qualitative analysis.mp. or exp qualitative analysis/

**32** 19 and 31

**31** 20 or 21 or 22 or 23 or 24 or 25 or 26 or 27 or 28 or 29 or 30

**30** Engagement.mp.

**29** Uptake.mp.

**28** Referral.mp. or patient referral/

**27** Accessibility.mp.

**26** Feasibility.mp.

**25** challenge$.mp.

**24** Adherence factor$.mp.

**23** Determinant$.mp.

**22** Influencing factor$.mp.

**21** Facilitator$.mp.

**20** Barrier$.mp.

**19** 5 and 18

**18** 6 or 7 or 8 or 9 or 10 or 11 or 12 or 13 or 14 or 15 or 16 or 17

**17** Influenza vaccination.mp. or exp influenza vaccination/

**16** Vaccination.mp. or exp vaccination/

**15** Influenza vaccine.mp. or influenza vaccine/

**14** Smoking cessation agent$.mp. or exp smoking cessation agent/

**13** Quitting smoking.mp.

**12** Smoking cessation.mp. or exp smoking cessation/

**11** Pulmonary rehabilitation.mp. or exp pulmonary rehabilitation/

**10** LTOT.mp.

**9** exp oxygen therapy/ or long term oxygen therapy.mp.

**8** Inhaler$ adherence.mp.

**7** Treatment adherence.mp.

**6** Medication adherence.mp. or exp medication compliance/

**5** 1 or 2 or 3 or 4

**4** Chronic obstructive pulmonary disease$.mp.

**3** COPD.mp.

**2** Chronic obstructive lung disease$.mp.

**1** Chronic obstructive pulmonary disease.mp. or exp chronic obstructive lung disease/

Scopus:

"Chronic obstructive pulmonary disease*" OR copd OR " chronic obstructive lung disease*"

AND

"medication adherence" OR "inhaler* adherence" OR "long term oxygen therapy" OR LTOT OR "pulmonary rehabilitation" OR "smoking cessation" OR "quitting smoking" OR "influenza vaccin*" OR "flu vaccine" OR "vaccin*"

AND

barrier* OR facilitator* OR "influence factor*" OR determinant* OR "adherence factor*" OR "challenge*" OR feasibility OR referral OR uptake OR engagement

AND

"qualitative analysis" OR " qualitative research" OR "semi structured interview" OR "telephone interview" OR interview* OR "focus group*" OR "mixed method study" OR "thematic Analysis" OR "qualitative study" OR "mixed method*"
