# Supplementary material for: Barriers and facilitators of adherence to treatment interventions for COPD amongst individuals from minority ethnic communities: Meta-ethnography
Source: PLoS One. 2025 Feb 10;20(2):e0318709. doi: 10.1371/journal.pone.0318709 (PMC11809908; doi:10.1371/journal.pone.0318709)
Supplement: S3 Table — (DOCX) [file pone.0318709.s003.docx]

**S3 Table: A numbered table of all 639 studies identified in the literature search:**

| **No** | **Study title** | **Reason for exclusion** |
| --- | --- | --- |
| 1 | Describing drivers of and barriers to persistence with tiotropium in patients with chronic obstructive pulmonary disease: A mixed-methods approach | No data on ethnicity provided |
| 2 | Vaccination coverage of recommended vaccines and determinants of vaccination in at-risk groups | No data on ethnicity provided |
| 3 | Experiences in elderly people with chronic obstructive pulmonary disease in relation to the use of long-term home oxygen therapy: a qualitative study about feelings attributed to therapy | No data on ethnicity provided |
| 4 | Implementation challenges in delivering team-based care ('TEAMcare') for patients with chronic obstructive pulmonary disease in a public hospital setting: a mixed methods approach | No data on ethnicity provided |
| 5 | Participant experiences of a community-based maintenance program post-pulmonary rehabilitation | No data on ethnicity provided |
| 6 | Improving Quality of Care for Patients with Chronic Obstructive Pulmonary Disease | No data on ethnicity provided |
| 7 | COPD: understanding patients’ adherence to inhaled medications | No data on ethnicity provided |
| 8 | Association of psychological flexibility with engagement in pulmonary rehabilitation following an acute exacerbation of chronic obstructive pulmonary disease | No data on ethnicity provided |
| 9 | Participation and drop-out in pulmonary rehabilitation: A qualitative analysis of the patient's perspective | No data on ethnicity provided |
| 10 | The lay health worker-patient relationship in promoting pulmonary rehabilitation (PR) in COPD: What makes it work? | No data on ethnicity provided |
| 11 | A personalized biomedical risk assessment infographic for people who smoke with COPD: a qualitative study | No data on ethnicity provided |
| 12 | Improving the uptake of pulmonary rehabilitation in patients with COPD: qualitative study of experiences and attitudes | No data on ethnicity provided |
| 13 | An Evaluation of Factors That Influence Referral to Pulmonary Rehabilitation Programs Among People With COPD | No data on ethnicity provided |
| 14 | A Qualitative Study to Inform a More Acceptable Pulmonary Rehabilitation Program after Acute Exacerbation of Chronic Obstructive Pulmonary Disease | No data on ethnicity provided |
| 15 | Which chronic obstructive pulmonary disease care recommendations have low implementation and why? A pilot study | No data on ethnicity provided |
| 16 | Lack of perceived benefit and inadequate transport influence uptake and completion of pulmonary rehabilitation in people with chronic obstructive pulmonary disease: A qualitative study | No data on ethnicity provided |
| 17 | Understanding the influences of copd patient's capability on the uptake of pulmonary rehabilitation in the uk through an inclusive design approach | No data on ethnicity provided |
| 18 | A qualitative assessment of COPD patients' experiences of pulmonary rehabilitation and guidance by healthcare professionals | No data on ethnicity provided |
| 19 | Barriers and motivational factors towards physical activity in daily life living with COPD-an interview based pilot study | No data on ethnicity provided |
| 20 | Developing Appropriate Pulmonary Rehabilitation Services in Sri Lanka: Assessment of People Living with COPD and Healthcare Providers in Urban and Semi Urban Areas in Sri Lanka | No data on ethnicity provided |
| 21 | Patients' and providers' perceptions of the impact of health literacy on communication in pulmonary rehabilitation | No data on ethnicity provided |
| 22 | Exploring the barriers to pulmonary rehabilitation for patients with chronic obstructive pulmonary disease: a qualitative study | No data on ethnicity provided |
| 23 | The Influence of Pulmonary Rehabilitation and Counselling on Perceptions of Physical Activity in Individuals with COPD – A Qualitative Stud | No data on ethnicity provided |
| 24 | Needs assessment for introducing pulmonary rehabilitation for chronic obstructive pulmonary disease management in a rural Indian setting: a qualitative study | No data on ethnicity provided |
| 25 | Barriers to and enablers of physical activity in patients with COPD following a hospital admission: A qualitative study | No data on ethnicity provided |
| 26 | A mixed methods study of seasonal influenza vaccine hesitancy in adults with chronic respiratory conditions | No data on ethnicity provided |
| 27 | A psychological intervention for smoking cessation delivered as treatment for smokers with chronic obstructive pulmonary disease: Multiple needs of a complex group and recommendations for novel service development | No data on ethnicity provided |
| 28 | Development of a web-based tool built from pharmacy claims data to assess adherence to respiratory medications in primary care | No data on ethnicity provided |
| 29 | Barriers and Facilitators for Smoking Cessation in Chinese Smokers with Chronic Obstructive Pulmonary Disease: A Qualitative Study | No data on ethnicity provided |
| 30 | Experiences of individuals using a novel web‑based rehabilitation programme: Self-management Programme of Activity Coping and Education (SPACE) for chronic obstructive pulmonary disease | No data on ethnicity provided |
| 31 | Using Exploratory Focus Groups to Inform the Development of a Peer-Supported Pulmonary Rehabilitation Program | No data on ethnicity provided |
| 32 | …But Watch Out for the Weather: Factors affecting adherence to progressive resistance exercise for persons with COPD | No data on ethnicity provided |
| 33 | Maintenance of a Physically Active Lifestyle After Pulmonary Rehabilitation in Patients With COPD: A Qualitative Study Toward Motivational Factors | No data on ethnicity provided |
| 34 | A telephone-based survey of current trends, habits and beliefs in patients receiving portable oxygen therapy in Madrid, Spain | No data on ethnicity provided |
| 36 | Adherence to pulmonary rehabilitation: A qualitative study | No data on ethnicity provided |
| 37 | The effects of a video intervention on posthospitalization pulmonary rehabilitation uptake | No data on ethnicity provided |
| 38 | Feasibility of a Health Coaching and Home-Based Rehabilitation Intervention With Remote Monitoring for COPD | No data on ethnicity provided |
| 39 | Barriers and Enablers to Pulmonary Rehabilitation in Low- and Middle-Income Countries: A Qualitative Study of Healthcare Professionals | Population not relevant (HCPs) |
| 40 | Signs of progress in the Australian post-2000 COPD experience, but some old problems remain | No data on ethnicity provided |
| 41 | Comparison of patient perceptions of Telehealth-supported and specialist nursing interventions for early stage COPD: a qualitative study | No data on ethnicity provided |
| 42 | 'It is not going to change his life but it has picked him up': A qualitative study of perspectives on long term oxygen therapy for people with chronic obstructive pulmonary disease | No data on ethnicity provided |
| 43 | Living in a rural area with advanced chronic respiratory illness: a qualitative study | No data on ethnicity provided |
| 44 | COPD transitions in health and self-management: Service users' experiences from everyday life | No data on ethnicity provided |
| 45 | Patients' experiences of health transitions in pulmonary rehabilitation | No data on ethnicity provided |
| 46 | 'Belonging'. 'Patients' experiences of social relationships during pulmonary rehabilitation | No data on ethnicity provided |
| 47 | Providing reviews of evidence to COPD patients: qualitative study of barriers and facilitating factors to patient-mediated practice change | No data on ethnicity provided |
| 48 | Adherence and factors affecting satisfaction in long-term telerehabilitation for patients with chronic obstructive pulmonary disease: a mixed methods study | No data on ethnicity provided |
| 49 | Patient acceptance of a telemedicine service for rehabilitation care: A focus group study | No data on ethnicity provided |
| 50 | A Virtual Reality-Supported Intervention for Pulmonary Rehabilitation of Patients With Chronic Obstructive Pulmonary Disease: Mixed Methods Study | No data on ethnicity provided |
| 51 | How integrated are services for patients with chronic obstructive pulmonary disease? Perceptions of patients and health care providers | No data on ethnicity provided |
| 52 | Barriers and enablers of physical activity engagement for patients with COPD in primary care | No data on ethnicity provided |
| 53 | Home-based pulmonary rehabilitation for people with COPD: A qualitative study reporting the patient perspective | No data on ethnicity provided |
| 54 | Uncertainty prior to pulmonary rehabilitation in primary care: A phenomenological qualitative study in patients with chronic obstructive pulmonary disease | No data on ethnicity provided |
| 55 | Feasibility of an online platform delivery of pulmonary rehabilitation for individuals with chronic respiratory disease | No data on ethnicity provided |
| 56 | Eenance of non-pharmacological strategies 6 months after patients with chronic obstructive pulmonary disease (COPD) attend a breathlessness service: A qualitative study | No data on ethnicity provided |
| 57 | Community-based exercise training for people with chronic respiratory and chronic cardiac disease: A mixed-methods evaluation | No data on ethnicity provided |
| 58 | Disease self-management in patients with moderate COPD: a thematic analysis | No data on ethnicity provided |
| 59 | Is inspiratory muscle training (IMT) an acceptable treatment option for people with chronic obstructive pulmonary disease (COPD) who have declined pulmonary rehabilitation (PR) and can IMT enhance PR uptake? A single-group prepost feasibility study in a home-based setting | No data on ethnicity provided |
| 60 | Clinician-Facilitated Physical Activity Intervention Versus Pulmonary Rehabilitation for Improving Physical Activity in COPD: A Feasibility Study | No data on ethnicity provided |
| 61 | Community lung health service design for COPD patients in China by the Breathe Well group | No data on ethnicity provided |
| 62 | Development and pretesting of a new functional-based health literacy measurement tool for chronic obstructive pulmonary disease (COPD) and asthma management | No data on ethnicity provided |
| 63 | Swimming pool-based exercise as pulmonary rehabilitation for COPD patients in primary care: Feasibility and acceptability | No data on ethnicity provided |
| 64 | Raised illness mastering - a phenomenological hermeneutic study of chronic obstructive pulmonary disease patients' experiences while participating in a long-term telerehabilitation programme | No data on ethnicity provided |
| 65 | Listen to Me! - A Mixed-Methods Study of Thoughts and Attitudes Towards Participation in Pulmonary Telerehabilitation Among People with Severe and Very Severe COPD Who Declined Participation in Pulmonary Rehabilitation | No data on ethnicity provided |
| 66 | Why do people with chronic obstructive pulmonary disease repeat pulmonary rehabilitation? Perspectives of patients and health professionals | No data on ethnicity provided |
| 67 | COPD patients' experience of long-term domestic oxygen-enriched nasal high flow treatment: A qualitative study | No data on ethnicity provided |
| 68 | Why do patients decline to take part in a research project involving pulmonary rehabilitation? | No data on ethnicity provided |
| 69 | Early home-based pulmonary rehabilitation following acute exacerbation of COPD: A feasibility study using an action research approach | No data on ethnicity provided |
| 70 | “I don’t mind damaging my own body” A qualitative study of the factors that motivate smokers to quit | No data on ethnicity provided |
| 71 | Barriers to adherence to home-based pulmonary rehabilitation among patients with chronic obstructive pulmonary disease in Iran: a descriptive qualitative study | No data on ethnicity provided |
| 72 | A home-based pulmonary rehabilitation mHealth system to enhance the exercise capacity of patients with COPD: development and evaluation | No data on ethnicity provided |
| 73 | Perceptions and experiences of a manual therapy trial: a qualitative study of people with moderate to severe COPD | No data on ethnicity provided |
| 74 | Evaluation of the Implementation of a Home-Based Exercise Training Program for People With COPD: A Mixed-Methods Study | No data on ethnicity provided |
| 75 | [Integrating Home-Based Exercise Training with a Hospital at Home Service for Patients Hospitalised with Acute Exacerbations of COPD: Developing the Model Using Accelerated Experience-Based Co-Design](https://libsearch.ncl.ac.uk/primo-explore/fulldisplay?docid=RS_6117691061035edexperiencebasedcodesign&context=SP&vid=NEWUI&lang=en_US) | No data on ethnicity provided |
| 76 | Behavioural modes of adherence to inspiratory muscle training in people with chronic obstructive pulmonary disease: a grounded theory study | No data on ethnicity provided |
| 77 | Reasons for missed appointments linked to a public-sector intervention targeting patients with stable chronic conditions in South Africa: results from in-depth interviews and a retrospective review of medical records | No data on ethnicity provided |
| 78 | Experiences of self-blame and stigmatisation for self-infliction among individuals living with COPD | No data on ethnicity provided |
| 79 | Facilitators and barriers to exercise maintenance in chronic obstructive pulmonary disease: patient views | No data on ethnicity provided |
| 80 | Internet-enabled pulmonary rehabilitation and diabetes education in group settings at home: a preliminary study of patient acceptability | No data on ethnicity provided |
| 81 | “We are not worthy” – understanding why patients decline pulmonary rehabilitation following an acute exacerbation of COPD | No data on ethnicity provided |
| 82 | Patients with COPD: Exploring patients’ coping ability during an interdisciplinary pulmonary rehabilitation programme: A qualitative focus group study | No data on ethnicity provided |
| 83 | Assessment of Adherence and Common Non-adherence Factors for Inhaled Medications in Asthma and Chronic Obstructive Pulmonary Disease (COPD) Patients | No data on ethnicity provided |
| 84 | Does Hospitalization Influence Patients’ Medication Adherence and Community Pharmacists’ Interventions? | No data on ethnicity provided |
| 85 | Living with chronic obstructive pulmonary disease: insiders’ perspectives | No data on ethnicity provided |
| 86 | from the perspective ofpatients with chronic obstructive pulmonary disease | No data on ethnicity provided |
| 87 | Illness experiences of persons with chronic obstructive pulmonary disease: self-perceived efficacy of home-based pulmonary rehabilitation | No data on ethnicity provided |
| 88 | Frequency of referral to and attendance at a pulmonaryrehabilitation programme amongst patients admitted to atertiary hospital with chronic obstructive pulmonary disease | No data on ethnicity provided |
| 89 | Service user perspectives on engagement in an occupational therapy-led pulmonary rehabilitation programme: A qualitative interview study | No data on ethnicity provided |
| 90 | How to Ensure Referral and Uptake for COPD Rehabilitation – Part 2: A Case of Integrated Care on How to Translate Findings of Cross- Sectorial Workflow to Improve Cross-Sectorial Rehabilitation | No data on ethnicity provided |
| 91 | Determinants of Sedentary Behaviour in Individuals with COPD: A QualitativeExploration Guided by the Theoretical Domains Framework | No data on ethnicity provided |
| 92 | Advance care planning education in pulmonary rehabilitation: A qualitative study exploring participant perspectives | No data on ethnicity provided |
| 93 | A Qualitative Exploration of Exercise Among Pulmonary Rehabilitation Participants: Insight From Multiple Sources of Social Influence | No data on ethnicity provided |
| 94 | The feasibility of early pulmonary rehabilitation and activity after COPD exacerbations: external pilot randomised controlled trial, qualitat | No data on ethnicity provided |
| 95 | A qualitative assessment of the pulmonary rehabilitation decision-making needs of patients living with COPD | All participants were white British |
| 96 | Barriers to smoking cessation: a qualitative study from the perspective of primary care in Malaysia | No quotations from minority ethnic groups |
| 97 | Explaining adherence to supplemental oxygen therapy: The patient's perspective | No quotations from minority ethnic groups |
| 98 | Feasibility of a Virtual Reality App to Promote Pulmonary Rehabilitation | Intervention not relevant |
| 99 | Smoking cessation: COPD patients' perspective | All participants were Caucasians |
| 100 | Improving COPD Care in a Medically Primary Care Clinic: A Qualitative Study of Patient Perspectives | Included |
| 101 | Improving understanding of and adherence to pulmonary rehabilitation in patients with COPD: A qualitative inquiry of patient and health professional perspectives | No quotations from minority ethnic groups |
| 102 | People with COPD perceive ongoing, structured and socially supportive exercise opportunities to be important for maintaining an active lifestyle following pulmonary rehabilitation: a qualitative study | Intervention not relevant |
| 103 | Perceptions and experiences of older patients and healthcare professionals regarding shared decision-making in pulmonary rehabilitation: A qualitative study | They mentioned that all participants from same ethnic group with no further details. |
| 104 | Participants' experiences of the benefits, barriers and facilitators of attending a community-based exercise programme for people with chronic obstructive pulmonary disease | All participants were Whites |
| 105 | Barriers and facilitators of self-management behaviors among patients with chronic obstructive pulmonary disease and chronic comorbidities: A mixed-methods investigation | No quotations from minority ethnic groups |
| 106 | Understanding barriers to and strategies for medication adherence in COPD: a qualitative study | No quotations from minority ethnic groups |
| 107 | Adherence to Pulmonary Rehabilitation in COPD: A QUALITATIVE EXPLORATION of PATIENT PERSPECTIVES on BARRIERS and FACILITATORS | No quotations from minority ethnic groups |
| 108 | Smoking Cessation in Pulmonary Care Subjects: A Mixed Methods Analysis of Treatment-Seeking Participation and Preferences | No quotations from minority ethnic groups |
| 109 | Application of the RE-AIM framework to evaluate the implementation of telehealth pulmonary rehabilitation in a randomized controlled trial among African-American and Hispanic patients with advanced stage Chronic Obstructive Pulmonary Disease | Included |
| 110 | An exploration of the smoking-related health beliefs of older people with chronic obstructive pulmonary disease | No quotations from minority ethnic groups |
| 111 | "You leave there feeling part of something": A qualitative study of hospitalized COPD patients' perceptions of pulmonary rehabilitation | No quotations from minority ethnic groups |
| 112 | Improving access to health care for people with severe chronic obstructive pulmonary disease (COPD) in Southern New Zealand: Qualitative study of the views of health professional stakeholders and patients | No quotations from minority ethnic groups |
| 113 | Specifications and feasibility of technology-based self-management of COPD: An exploratory qualitative study with patients and providers | All participants were Whites |
| 114 | Experiences of pulmonary rehabilitation in people living with chronic obstructive pulmonary disease and frailty A qualitative interview study | Included |
| 115 | A prospective qualitative exploration of views about attending pulmonary rehabilitation | All participants were Caucasians |
| 116 | Pulmonary rehabilitation referral and uptake from primary care for people living with COPD: A mixed-methods study | Included |
| 117 | Whakawhanaungatanga: The importance of culturally meaningful connections to improve uptake of pulmonary rehabilitation by maori with COPD - A qualitative study | Included |
| 118 | Giving Voice to People - Experiences During Mild to Moderate Acute Exacerbations of COPD | No data on ethnicity provided |
| 119 | Feasibility of a pulmonary rehabilitation programme for patients with symptomatic chronic obstructive pulmonary disease in Georgia: A single-site, randomised controlled trial from the Breathe Well Group | No quotations from minority ethnic groups |
| 120 | Sing Your Lungs Out: A qualitative study of a community singing group for people with chronic obstructive pulmonary disease (COPD) | No quotations from minority ethnic groups |
| 121 | Acceptability and feasibility of pulmonary rehabilitation for COPD: A community qualitative study | No quotations from minority ethnic groups |
| 122 | Photovoice exploration of physical activity norms and values among rural and remote pulmonary rehabilitation participants in British Columbia, Canada | No data on ethnicity provided |
| 123 | Use of Information and Communication Technology among Patients with Chronic Obstructive Pulmonary Disease Who Smoke | No quotations from minority ethnic groups |
| 124 | Stakeholder Perceptions of a Web-Based Physical Activity Intervention for COPD: A Mixed-Methods Study | No quotations from minority ethnic groups |
| 125 | The primary care experience of adults with chronic obstructive pulmonary disease (COPD). An interpretative phenomenological inquiry | No quotations from minority ethnic groups |
| 126 | A Telehealth-Delivered Pulmonary Rehabilitation Intervention in Underserved Hispanic and African American Patients With Chronic Obstructive Pulmonary Disease: A Community-Based Participatory Research Approach | Included |
| 127 | Evaluation of ‘care bundles’ for patients with chronic obstructive pulmonary disease (COPD): a multisite study in the UK | Intervention not relevant |
| 128 | Patient Involvement in the Design of a Patient-Centered Clinical Trial to Promote Adherence to Supplemental Oxygen Therapy in COPD | No quotations from minority ethnic groups |
| 129 | Why do Chinese people with COPD continue smoking: the attitudes and beliefs of Chinese residents of Vancouver, Canada | Included |
| 130 | Developing a home-based pulmonary rehabilitation programme for patients with chronic respiratory diseases in Malaysia: A mixed-method feasibility study | No quotations from minority ethnic groups |
| 131 | Investigating primary health care practitioners’ barriers and enablers to referral of COPD patients to Pulmonary Rehabilitation: an exploratory sequential mixed methods study using the Theoretical Domains Framework | Population not relevant (HCPs) |
| 132 | Life Impact and Treatment Preferences of Individuals with Asthma and Chronic Obstructive Pulmonary Disease: Results from Qualitative Interviews and Focus Groups | No quotations from minority ethnic groups |
| 133 | Attitude regarding advance directives among patients with pulmonary rehabilitation | Irrelevant |
| 134 | Using exploratory focus groups to inform the development of a peer-supported pulmonary rehabilitation program: Directions for further research | No data on ethnicity provided |
| 135 | Barriers to and facilitators of medication adherence | Irrelevant |
| 136 | Promoting Participation in Pulmonary Rehabilitation after Hospitalization for Chronic Obstructive Pulmonary Disease, Strategies of Top-performing Systems: A Qualitative Study | Population not relevant |
| 137 | Pragmatic Challenge of Sustainability: Long-Term Adherence to COPD Care Bundle Maintains Lower Readmission Rate | Irrelevant |
| 138 | Analysis of vaccination characteristics and influencing factors of influenza vaccine and pneumococcal vaccine in patients with frequent acute exacerbation phenotype COPD | No data on ethnicity provided |
| 139 | Home-Based Pulmonary Rehabilitation and Health Coaching in Fibrotic Interstitial Lung Disease: IMPLEMENTATION AND QUALITATIVE ASSESSMENT OF A PILOT TELEHEALTH PROGRAM | Irrelevant |
| 140 | Stable copd management in relation to the GOLD: Experience at a university hospital | Irrelevant |
| 141 | The development of a home-based respiratory rehabilitation program | Irrelevant |
| 142 | Problematic Activities of Daily Life are Weakly Associated With Clinical Characteristics in COPD | Irrelevant |
| 143 | Exploring the Views of Individuals With Chronic Obstructive Pulmonary Disease on the Use of Rollators | Irrelevant |
| 144 | Strategies to Improve Enrollment and Participation in Pulmonary Rehabilitation Following a Hospitalization for COPD | Irrelevant |
| 145 | Identifying the barriers to pulmonary rehabilitation for patients with COPD | Not published in English |
| 146 | Analysis on respiratory rehabilitation in patients with chronic obstructive pulmonary disease aged 40 years or older in China, 2014-2015 | Not published in English |
| 147 | Mental barriers to quitting smoking: The association between smoking and depression in COPD patients. | Not published in English |
| 148 | COPD and smoking cessation: Patients' expectations and responses of health professionals | Not published in English |
| 149 | Tobacco dependence status and influencing factors among smokers aged 40 or older in China. | Not published in English |
| 150 | White P. Acceptability and feasibility of pulmonary rehabilitation for COPD: A community qualitative study. | Not published in English |
| 151 | Influenza and pneumococcal vaccination uptake in adults aged >=65 years and high risk groups admitted to yozgat bozok university research and application hospital | Not published in English |
| 152 | Barreiras à adesão ao programa de reabilitação pulmonar de pacientes com doença pulmonar obstrutiva crônica | Not published in English |
| 153 | Physicians and Nurses' Perspective of Barriers and Solutions to the Delivery of Home-based Pulmonary Rehabilitation for Patients with Chronic Obstructive Pulmonary Disease：a Qualitative Study | Not published in English |
| 154 | Chronic Obstructive Pulmonary Disease in Eastern Black Sea Region: Characteristics of the Disease and the Frequency of Influenza-Pneumococcal Vaccination | Not published in English |
| 155 | Shared decision making in pulmonary rehabilitation: A qualitative needs assessment | Conference abstract |
| 156 | Social network size and social participation amongst community dwelling patients with COPD: A mixed methods study | Conference abstract |
| 157 | Australian perceptions of pertussis and vaccine preventable disease in the setting of asthma and COPD | Conference abstract |
| 158 | Barriers to early diagnosis and smoking cessation for COPD patients | Conference abstract |
| 159 | Mental health diagnosis impacts choice of smoking cessation therapy in veterans with chronic obstructive pulmonary disease | Conference abstract |
| 160 | Video testimonials may facilitate understanding of and referral to pulmonary rehabilitation in people with chronic respiratory disease | Conference abstract |
| 161 | Barriers to exercise following pulmonary rehabilitation in individuals with COPD: A mixed methods study | Conference abstract |
| 162 | Detriments to Pulmonary Rehabilitation Referral in a Rural Community Hospital: The Qi Project | Conference abstract |
| 163 | Barriers to smoking cessation: The patient's perspective | Conference abstract |
| 164 | Enablers and barriers in referral and uptake of pulmonary rehabilitation (PR) in a south Asian patient group with COPD: A qualitative study | Conference abstract |
| 165 | Lay health workers in pulmonary rehabilitation-recruitment and training of COPD patient volunteers | Conference abstract |
| 166 | Patient's perception of pulmonary rehabilitation in Bangladesh | Conference abstract |
| 167 | Referral to telehealth delivered pulmonary rehabilitation (TelePR) versus standard pulmonary rehabilitation (SPR) in hispanic and african patients hospitalized for COPD Exacerbations: Results of a randomized controlled trial | Conference abstract |
| 168 | Providing pulmonary rehabilitation to individuals from CALD backgrounds | Conference abstract |
| 169 | Home-based and hospital-based pulmonary rehabilitation in patients with COPD-does the location influence completion rates? | Conference abstract |
| 170 | SPACE for COPD: Experiences of using a web based rehabilitation programme | Conference abstract |
| 171 | Respiratory clinic referral patterns to an outpatient pulmonary rehabilitation program | Conference abstract |
| 172 | Relationship between perceived medication adherence barriers and incentive preference | Conference abstract |
| 173 | Beliefs about medicines in obstructive pulmonary diseases | Conference abstract |
| 174 | Predictors of influenza vaccination in patients with chronic obstructive pulmonary disease: Analysis of the 2012 behavioral risk factors surveillance system | Conference abstract |
| 175 | Setting the stage for a more acceptable intervention to patients: Patients' and healthcare professionals' views on the delivery of pulmonary rehabilitation post-acute exacerbation of COPD | Conference abstract |
| 176 | Pulmonary rehabilitation in the acute and community setting: What's happening and where | Conference abstract |
| 177 | Investigation into patient attitudes and barriers to influenza vaccination in a cohort of high-risk patients attending respiratory outpatient clinics | Conference abstract |
| 178 | Driving Forces for Adherence to Low-Intensity Pulmonary Rehabilitation and Exercise among in Adults with COPD Receiving Care in a Resource-Limited Setting | Conference abstract |
| 179 | COPD patients' beliefs and expectations of pulmonary rehabilitation | Conference abstract |
| 180 | Barriers and facilitators of self-management behaviors among patients with multiple chronic conditions: A mixed-Methods investigation | Conference abstract |
| 181 | Attitudes, beliefs and organizational barriers regarding safe emergency oxygen therapy for patients with COPD: A mixed methods study | Conference abstract |
| 182 | Predictors and barriers to uptake of influenza vaccination among African American patients with chronic obstructive pulmonary disease in a large academic hospital | Conference abstract |
| 183 | Patient perspectives and barriers to pulmonary rehabilitation following hospitalizations for chronic obstructive pulmonary disease exacerbations | Conference abstract |
| 184 | Physical activity, exercise capacity and psycho-social factors during and after pulmonary rehabilitation in COPD | Conference abstract |
| 185 | Barriers to the integration of tobacco cessation programs in primary care practices in Tennessee | Conference abstract |
| 186 | Barriers to the uptake and completion of pulmonary rehabilitation | Conference abstract |
| 187 | Patient and public involvement and application of the behaviour change wheel to promote physical activity following pulmonary rehabilitation in COPD: An intervention development study | Conference abstract |
| 188 | Patient Perceptions of a Web-Based Physical Activity Intervention for COPD: A Mixed- Methods Study | Conference abstract |
| 189 | Real world utilization of pulmonary rehabilitation after an acute exacerbation of COPD; a retrospective study of patients who attended a COPD exacerbation clinic | Conference abstract |
| 200 | Improving Completion to Pulmonary Rehabilitation: A Pilot Study | Conference abstract |
| 201 | Analysis of pulmonary rehabilitation (Pr) treatment patterns and utilization in patients with chronic obstructive pulmonary disease (COPD) in The United States (US) | Conference abstract |
| 202 | Understanding reasons for patient attendance and non-attendance in pulmonary rehabilitation and COPD self-management programmes. A qualitative synthesis and application of theory | Conference abstract |
| 203 | Hospitalized copd patients' perceptions of pulmonary rehabilitation: Barriers and facilitators to enrollment and adherence | Conference abstract |
| 204 | Modified interstitial lung disease (ILD) pulmonary rehabilitation (PR) programme: patients' perceptions | Conference abstract |
| 205 | A survey of uptake and barriers to the seasonal influenza vaccination in respirology patients at an ontario academic hospital | Conference abstract |
| 206 | Barriers and facilitators to low-intensity pulmonary rehabilitation in a 'safety-net' setting | Conference abstract |
| 207 | Real time qualitative primary-care based study related to the validity of the diagnosis and management of patients held on COPD registers in primary care | Conference abstract |
| 208 | The effectiveness of a Chinese pulmonary rehabilitation program | Conference abstract |
| 209 | Post-Hospitalisation Pulmonary Rehabilitation (PHPR) in COPD patients-A retrospective cohort study | Conference abstract |
| 210 | Understanding referral to pulmonary rehabilitation for COPD patients by Primary Health Care staff-a qualitative study using the Theoretical Domains Framework | Conference abstract |
| 211 | Supporting COPD patients to access pulmonary rehabilitation with lay health workers: A feasibility study | Conference abstract |
| 212 | The lay health worker-patient partnership promoting PR in COPD: What makes it work? | Conference abstract |
| 213 | Pulmonary rehabilitation utilization in a regional Australian health service | Conference abstract |
| 214 | Recommendations for smoking cessation service provision for smokers with copd with multiple complex needs: Findings from a pilot study | Conference abstract |
| 215 | Pulmonary Rehabilitation Awareness and Uptake in an Engaged Cohort of People with Copd | Conference abstract |
| 216 | What patients report regarding chronic obstructive pulmonary disease medication adherence | Conference abstract |
| 217 | Sex Differences in Prevalence and Determinants of Anxiety in Patients with COPD Initiating Pulmonary Rehabilitation | Conference abstract |
| 218 | Barriers and Motivators for Smoking Cessation in Chinese Patients With Chronic Obstructive Pulmonary Disease | Conference abstract |
| 219 | COPD patients derived benefits from attending PR: 'This has given me my life back' | Conference abstract |
| 220 | [Acute exacerbations of chronic obstructive pulmonary disease (AE-COPD): A qualitative exploratory study of patients' needs following discharge](https://libsearch.ncl.ac.uk/primo-explore/fulldisplay?docid=RS_609031936Alkhathlan%20Bsneedsfollowingdischarge&context=SP&vid=NEWUI&lang=en_US) | Conference abstract |
| 221 | No single system of pulmonary rehabilitation delivers for all patients with COPD ? | Conference abstract |
| 222 | Home-based exercise training(HET) post-hospitalisation for acute exacerbation of COPD(AECOPD) - a mixed-method systematic review | Conference abstract |
| 223 | Exploring beliefs of Pulmonary Rehabilitation modes of delivery | Conference abstract |
| 224 | [Patients' perception on long-term domiciliary oxygen therapy](https://libsearch.ncl.ac.uk/primo-explore/fulldisplay?docid=RS_609031936Bueno%20GHmdomiciliaryoxygentherapy&context=SP&vid=NEWUI&lang=en_US) | Conference abstract |
| 225 | Factors associated with non-attendance to pulmonary rehabilitation in Canterbury, New Zealand | Conference abstract |
| 226 | Patient Preference for Chronic Obstructive Pulmonary Disease (Copd) Treatment Inhalers: A Discrete Choice Experiment (Dce) in France | Conference abstract |
| 227 | Assessment of self-management needs among patients with COPD | Conference abstract |
| 228 | Attitudes to online delivery of health information and chronic disease management in chronic obstructive pulmonary disease: Focus group study | Conference abstract |
| 229 | Attitudes to cognitive impairment and testing in patients with chronic obstructive pulmonary disease: Focus group study | Conference abstract |
| 230 | Understanding utilisation of pulmonary rehabilitation in primary care: An online survey | Conference abstract |
| 231 | Improving referral and uptake to pulmonary rehabilitation in primary care: Qualitative findings from an online survey in primary care | Conference abstract |
| 232 | Patients' experiences of early post-hospitalisation pulmonary rehabilitation: A quality improvement initiative | Conference abstract |
| 233 | Time trends (2012-2020) and sex differences for influenza vaccination uptake among individuals with chronic obstructive pulmonary disease in Spain | Conference abstract |
| 234 | Perceptions of patients about Pulmonary Rehabilitation (PR)in Bangladesh | Conference abstract |
| 235 | Participants perspectives of pulmonary rehabilitation: The role of peer support | Conference abstract |
| 236 | Chronic obstructive pulmonary disease: Adherence difficulties and suggestions from patients with home oxygen therapy | Conference abstract |
| 237 | Half of COPD patients appropriate for pulmonary rehab were referred | Conference abstract |
| 238 | Illness and social factors influence attrition in pulmonary rehabilitation | Conference abstract |
| 239 | Living with respiratory illness in rural Saskatchewan: A qualitative pilot study | Conference abstract |
| 240 | Physician knowledge and perception of COPD management in Korea and Japan: Continuing to confront COPD (C2C) physician survey 2012-2013 | Conference abstract |
| 241 | Post-hospitalisation outpatient pulmonary rehabilitation: A translational gap? | Conference abstract |
| 242 | Facilitation of continued exercise via patient volunteers with chronic obstructive pulmonary disease (COPD) following a pulmonary rehabilitation programme: A feasibility study | Conference abstract |
| 243 | The patient perspective on challenges to participating in pulmonary rehabilitation: An international web-based survey | Conference abstract |
| 244 | Factors affecting the dietary intake of people on home oxygen therapy (HOT) due to chronic obstructive pulmonary disease | Conference abstract |
| 245 | Factors affecting patients compliance with ambulatory oxygen therapy | Conference abstract |
| 246 | Virtual pulmonary rehabilitation programme - A new era of working | Conference abstract |
| 247 | Suboptimal Uptake of Outpatient Pulmonary Rehabilitation Following Discharge for a Copd Exacerbation: One Hospital's Experience | Conference abstract |
| 248 | Referrals to pulmonary rehabilitation after acute exacerbations of COPD: A mixed-methods evaluation | Conference abstract |
| 249 | Can a 6 week multi-disciplinary weekend pulmonary rehabilitation service for patients with weekday work commitments be both feasible and effective? | Conference abstract |
| 250 | Adapting telehealth pulmonary rehabilitation program to meet the needs of hispanic and African-American patients from disparity communities: A community-based participatory research approach | Conference abstract |
| 251 | Development of Culturally Appropriate Pulmonary Rehabilitation (PR)for Sri Lanka: A qualitative study | Conference abstract |
| 252 | Group-based social identity intervention during pulmonary rehabilitation improves COPD patients experience and promotes compliance with exercise programme | Conference abstract |
| 253 | Lung health of opiate users (LHOP): A pilot study to assess the respiratory health of opiate misusers attending a community substance misuse clinic | Conference abstract |
| 254 | The impact of telehealth-delivered pulmonary rehabilitation on social isolation in hispanic and african-american copd patients | Conference abstract |
| 255 | [Improving care for patients with chronic obstructive pulmonary disease in an urban hospital outpatient clinic](https://libsearch.ncl.ac.uk/primo-explore/fulldisplay?docid=RS_61073449Reardon%20JZnhospitaloutpatientclinic&context=SP&vid=NEWUI&lang=en_US) | Conference abstract |
| 256 | From face-to-face to telerehabilitation: patients prefer a mixed model on a pandemic-free future | Conference abstract |
| 257 | Response shift in COPD patients undertaking pulmonary rehabilitation | Conference abstract |
| 258 | Experiences of training-adherence in a 12 weeks home-based IMT program for individuals with COPD | Conference abstract |
| 259 | Social Determinants of Adherence to Pulmonary Rehabilitation in Chronic Obstructive Pulmonary Disease: A Mixed Methods Study | Conference abstract |
| 260 | REFERRALS TO PULMONARY REHABILITATION AFTER ACUTE EXACERBATIONS OF COPD: A MIXED-METHODS EVALUATION | Conference abstract |
| 261 | Implementation of offering choice of pulmonary rehabilitation location to people with COPD: a protocol for the process evaluation of a cluster randomised controlled trial | Protocol |
| 263 | Smoking knowledge, attitudes, practice and behavior in outpatient individuals with COPD in zunyi city, china | Protocol |
| 264 | Medication adherence and patient-reported outcomes in subjects with chronic obstructive pulmonary disease | Protocol |
| 265 | Home-based pulmonary rehabilitation in chronic obstructive pulmonary disease: The patient experience | Protocol |
| 266 | Evaluation of compliance of pulmonary rehabilitation program in COPD patients | Protocol |
| 267 | Recruiting adult offspring of people with COPD to explore intergenerational lung health: Feasibility study | Protocol |
| 268 | Factors influencing participation and adherence to a pulmonary rehabilitation program in patients with COPD: A qualitative study | Protocol |
| 269 | Which group of patients withdraw from a pulmonary rehabilitation program? | Protocol |
| 270 | Determinants of variance in oxygen consumption at peak exercise in COPD | Protocol |
| 271 | On TRACk: Through training, preparation, and counseling, to better use of inhaled medication | Protocol |
| 272 | A pulmonary rehabilitation shared decision-making intervention for patients living with COPD: PReSent: protocol for a feasibility study | Protocol |
| 273 | Feasibility of home-based telerehabilitation in older adults with chronic obstructive pulmonary disease | Protocol |
| 274 | Integrating comprehensive geriatric assessment for people with copd and frailty starting pulmonary rehabilitation: The breathe plus feasibility trial protocol | Protocol |
| 275 | What prevents people with chronic obstructive pulmonary disease from atttending pulmonary rehabilitation? | Conference abstract |
| 276 | OPTImising the implementation of pulMonary rehAbiLitation in people with chronic obstructive pulmonary disease (the OPTIMAL study): mixed methods study protocol | Review |
| 277 | Discrepancies in Predictive Factors of Inhaler Technique vs. Adherence | Review |
| 278 | Up-to-date guidance towards improving medication adherence in patients with chronic obstructive pulmonary disease | Review |
| 279 | Why Do Patients with COPD Decline Rehabilitation | Review |
| 280 | The relationship between physical activity and health status in patients with chronic obstructive pulmonary disease following pulmonary rehabilitation | Review |
| 281 | Status of and strategies for improving adherence to COPD treatment | Review |
| 282 | Improving Uptake of Pulmonary Rehabilitation after a Chronic Obstructive Pulmonary Disease Exacerbation | Review |
| 283 | Determinants of successful completion of pulmonary rehabilitation in COPD | Quantitative |
| 284 | Prospective determinants of smoking cessation in COPD patients within a high intensity or a brief counseling intervention | Quantitative |
| 285 | Satisfaction with chronic obstructive pulmonary disease treatment: results from a multicenter, observational study | Quantitative |
| 286 | Cigarette Smoking, Tooth Loss, and Chronic Obstructive Pulmonary Disease: Findings From the Behavioral Risk Factor Surveillance System | Quantitative |
| 287 | Effect of high-deductible health plans on healthcare access, financial strain, medication adherence, and outcomes for patients with COPD: Findings from the national health interview survey | Quantitative |
| 289 | The gap in use of bronchodilators, inhaled corticosteroids and influenza vaccine among 23 high and low income sites | Quantitative |
| 290 | Barriers to enrollment in pulmonary rehabilitation: medical knowledge analysis | Quantitative |
| 291 | Treatment Adherence in Patients with Obstructive Pulmonary Diseases | Quantitative |
| 292 | Patient perceptions of the adequacy of supplemental oxygen therapy: Results of the American thoracic society nursing assembly oxygen working group survey | Quantitative |
| 293 | Exploring variables associated with medication non-adherence in patients with COPD | Quantitative |
| 294 | The association of health literacy with illness and medication beliefs among patients with chronic obstructive pulmonary disease | Quantitative |
| 295 | Potential risk factors for medication non-adherence in patients with chronic obstructive pulmonary disease (COPD) | Quantitative |
| 296 | Gender and Age as Determinants of Success of Pulmonary Rehabilitation in Individuals With Chronic Obstructive Pulmonary Disease | Quantitative |
| 297 | Study on Utilization Pattern of Pulmonary Inhalers in Inpatient at Tertiary Care Teaching Hospital, Coimbatore, India | Quantitative |
| 298 | Failure to optimise stable COPD leads to hospital admission | Conference abstract |
| 299 | Association Between Unmet Essential Social Needs and Influenza Vaccination in US Adults | Quantitative |
| 300 | Smoking-cessation advice to patients with chronic obstructive pulmonary disease: The critical roles of health insurance and source of care | Quantitative |
| 301 | Differences in adherence barriers to inhaled medicines between japanese patients with chronic obstructive pulmonary disease and asthma evaluated using the "adherence starts with knowledge 20" (ASK-20) questionnaire | Quantitative |
| 302 | Medication adherence among patients with chronic obstructive pulmonary disease treated in a primary general hospital during the COVID-19 pandemic | Quantitative |
| 303 | Time Trends (2012-2020), Sex Differences and Predictors for Influenza Vaccination Uptake among Individuals with Chronic Obstructive Pulmonary Disease in Spain | Quantitative |
| 304 | Influenza vaccination coverage and uptake predictors among Spanish adults suffering COPD | Quantitative |
| 304 | Improving Influenza Vaccination Coverage Among Patients With COPD: A Pilot Project | Quantitative |
| 305 | Medication Adherence Assessment and Cost Analysis of COPD Treatment Under Out-Patient Clinic in Vietnam | Quantitative |
| 306 | High-Deductible Health Plans and Healthcare Access, Use, and Financial Strain in Those with Chronic Obstructive Pulmonary Disease | Quantitative |
| 307 | Adherence to COPD treatment in Turkey and saudi arabia: results of the aDCare study | Quantitative |
| 308 | Video Telehealth Pulmonary Rehabilitation for Chronic Obstructive Pulmonary Disease Is Associated with Clinical Improvement Similar to Center-based Pulmonary Rehabilitation | Irrelevant |
| 309 | Enhancing pulmonary rehabilitation enrollment study (PRESS III) | No data on ethnicity provided |
| 310 | Improving the uptake: Barriers and facilitators to pulmonary rehabilitation | Quantitative |
| 311 | Barriers and facilitators of medication adherence | Irrelevant |
| 312 | Perceptions and attitudes toward the use of nebulized therapy for COPD: Patient and caregiver perspectives | Intervention not relevant |
| 313 | Inhaler technique knowledge and skills before and after an educational program in obstructive respiratory disease patients: A real-life pilot study | No data on ethnicity provided |
| 314 | Perceptions of pulmonary rehabilitation by health professionals and culturally and linguistically diverse COPD patients | Population not relevant (HCPs) |
| 315 | Unmet need in the management of chronic obstructive pulmonary disease in the Middle East and Africa region: An expert panel consensus | Population not relevant (HCPs) |
| 316 | Healthcare providers' attitudes, beliefs and barriers to pulmonary rehabilitation for patients with chronic obstructive pulmonary disease in Saudi Arabia: A cross-sectional study | Population not relevant (HCPs) |
| 317 | Influenza vaccination for patients with chronic obstructive pulmonary disease: Implications for pharmacists | Population not relevant (HCPs) |
| 318 | A modified Delphi consensus study to identify improvement proposals for COPD management amongst clinicians and administrators in Spain | Population not relevant (HCPs) |
| 319 | Physician and patient perceptions in COPD: The COPD Resource Network Needs Assessment Survey | Population not relevant (HCPs) |
| 320 | Why don't our patients with chronic obstructive pulmonary disease listen to us? the enigma of nonadherence | Population not relevant (HCPs) |
| 320 | Barriers for delivering care to COPD patients: A physician survey | Population not relevant (HCPs) |
| 321 | Impact of a pharmacist-led, primary medication nonadherence intervention program on prescription fills in underserved patient populations | Population not relevant (HCPs) |
| 322 | Cliniciansa Perceived Barriers and Facilitators to Optimal Acute Oxygen Use | Population not relevant (HCPs) |
| 323 | Perception of caregivers on the care of patients with COPD in use of LTOT | Population not relevant (HCPs) |
| 324 | Challenges faced by rural primary care providers when caring for COPD patients in the Western United States | Population not relevant (HCPs) |
| 325 | Guideline-based COPD management in a resource-limited setting - physicians' understanding, adherence and barriers: A cross-sectional survey of internal and family medicine hospital-based physicians in Nigeria | Population not relevant (HCPs) |
| 326 | Adherence to Long-Acting Bronchodilators After Discharge for COPD: How Much of the Geographic Variation is Attributable to the Hospital of Discharge and How Much to the Primary Care Providers? | Population not relevant (HCPs) |
| 327 | TANDEM (Tailored intervention for anxiety and depression management in COPD) trial: Qualitative interviews with health care professionals from the pilot phase | Population not relevant (HCPs) |
| 328 | Developing a strategic understanding of telehealth service adoption for COPD care management: A causal loop analysis of healthcare professionals | Population not relevant (HCPs) |
| 329 | Challenges and opportunities for telehealth in the management of chronic obstructive pulmonary disease: a qualitative case study in Greece | Population not relevant (HCPs) |
| 330 | Barriers to enrollment in pulmonary rehabilitation: medical knowledge analysis | Population not relevant (HCPs) |
| 331 | Perceptions of Practitioners on Telehealth and App Use for Smoking Cessation and COPD Care-An Exploratory Study | Population not relevant (HCPs) |
| 332 | Public Health Practitioners' Knowledge towards Nicotine and Other Cigarette Components on Various Human Diseases in Pakistan: A Contribution to Smoking Cessation Policies | Population not relevant (HCPs) |
| 333 | Meeting the challenge of COPD care delivery in the USA: A multiprovider perspective | Population not relevant (HCPs) |
| 334 | Stakeholders' Views on Reducing Psychological Distress in Chronic Obstructive Pulmonary Disease | Population not relevant (HCPs) |
| 335 | Respiratory clinic referral patterns to an outpatient pulmonary rehabilitation program | Population not relevant (HCPs) |
| 336 | Primary Care Physicians, and Clinician's Knowledge, Attitudes and Beliefs Regarding Pulmonary Rehabilitation (A Focus on Africa) | Population not relevant (HCPs) |
| 337 | Perceptions of general practitioners regarding barriers and facilitators for referral to pulmonary rehabilitation | Population not relevant (HCPs) |
| 338 | Improving pulmonary rehabilitation referral rates from primary care physicians: Identifying barriers to referral | Population not relevant (HCPs) |
| 339 | Knowledge and Perception Towards Pulmonary Rehabilitation by Health Care Professionals and Barriers | Population not relevant (HCPs) |
| 340 | Intensive care unit nurses' perceptions of patient participation in the acute phase of chronic obstructive pulmonary disease exacerbation: an interview study | Population not relevant (HCPs) |
| 341 | Perspectives and Attitudes of General Practitioners Towards Pharmacological and Non-Pharmacological COPD Management in a Belgian Primary Care Setting: A Qualitative Study | Population not relevant (HCPs) |
| 342 | "Finding the Way in the Dark?- Working experience of specific-duty-post nurses in a nurse-led in-hospital pulmonary rehabilitation program: A qualitative study | Population not relevant (HCPs) |
| 343 | Enablers and barriers for implementing a COS for pulmonary rehabilitation in people with COPD-health professionals' perspectives | Population not relevant (HCPs) |
| 344 | Danish general practitioners' management of patients with COPD: a nationwide survey | Population not relevant (HCPs) |
| 345 | Barriers to COPD-patients' starting pulmonary rehabilitation: a comparison between telehealth and standard pulmonary rehabilitation referrals | Population not relevant (HCPs) |
| 346 | Psychosocial burden of caregivers is a hurdle for adherence to pulmonary rehabilitation program in a resource-scarce setting | Population not relevant (HCPs) |
| 347 | Perceptions and attitudes toward the use of nebulized therapy for COPD: Patient and caregiver perspectives | Population not relevant (HCPs) |
| 348 | The value of theory in programmes to implement clinical guidelines: Insights from a retrospective mixed-methods evaluation of a programme to increase adherence to national guidelines for chronic disease in primary care | Population not relevant (HCPs) |
| 349 | Exploring the barriers and facilitators for the use of digital health technologies for the management of COPD: a qualitative study of clinician perceptions | Population not relevant (HCPs) |
| 350 | Piecing together the jigsaw: Healthcare professionals' perceptions of pulmonary rehabilitation for patients with COPD | Population not relevant (HCPs) |
| 351 | Healthcare professionals (HCPs) attitudes towards referring patients to pulmonary rehabilitation (PR); a cross sectional exploratory survey | Population not relevant (HCPs) |
| 352 | What motivates primary healthcare practitioners to refer patients with Chronic Obstructive Pulmonary Disease (COPD) to Pulmonary Rehabilitation? A survey using the Theoretical Domains Framework | Population not relevant (HCPs) |
| 353 | Understanding referral to Pulmonary Rehabilitation for COPD patients by Primary Health Care staff - a qualitative study using the Theoretical Domains Framework | Population not relevant (HCPs) |
| 354 | Investigating primary healthcare practitioners' barriers and enablers to referral of patients with COPD to pulmonary rehabilitation: a mixed-methods study using the Theoretical Domains Framework | Population not relevant (HCPs) |
| 355 | The lay health worker-patient partnership promoting PR in COPD: What makes it work? | Population not relevant (HCPs) |
| 356 | Rural Primary Care Provider Perspectives on Barriers and Facilitators to Evidence-Based Management of Copd among Veterans | Population not relevant (HCPs) |
| 357 | Healthcare Professionals' Vaccine Recommendations in People with Copd | Population not relevant (HCPs) |
| 358 | Knowledge and attitudes of family physicians coming to COPD continuing medical education | Population not relevant (HCPs) |
| 359 | SPACE FOR COPD delivered as a maintenance programme on pulmonary rehabilitation discharge: protocol of a randomised controlled trial evaluating the long-term effects on exercise tolerance and mental well-being | Protocol |
| 360 | No single system of pulmonary rehabilitation delivers for all patients with COPD? | Conference abstract |
| 361 | Exploring beliefs of Pulmonary Rehabilitation modes of delivery | Conference abstract |
| 362 | Feasibility of home-based telerehabilitation in older adults with chronic obstructive pulmonary disease | Conference abstract |
| 363 | Factors associated with non-attendance to pulmonary rehabilitation in Canterbury, New Zealand | Conference abstract |
| 364 | Mindfulness-Based Programs for People with Chronic Obstructive Pulmonary Disease: a Mixed Methods Systematic Review | Review |
| 365 | Tobacco dependence status and influencing factors among smokers aged 40 or older in China. [Chinese] | Not published in English |
| 366 | Assessment of self-management needs among patients with COPD | Irrelevant |
| 367 | Attitudes to online delivery of health information and chronic disease management in chronic obstructive pulmonary disease: Focus group study | Irrelevant |
| 368 | Attitudes to cognitive impairment and testing in patients with chronic obstructive pulmonary disease: Focus group study | Irrelevant |
| 369 | Signs of progress in the Australian post-2000 COPD experience, but some old problems remain | Irrelevant |
| 370 | Understanding utilisation of pulmonary rehabilitation in primary care: An online survey | Conference abstract |
| 371 | Improving referral and uptake to pulmonary rehabilitation in primary care: Qualitative findings from an online survey in primary care | Conference abstract |
| 372 | Comparison of patient perceptions of Telehealth-supported and specialist nursing interventions for early stage COPD: a qualitative study | No data on ethnicity provided |
| 373 | A scoping review of co-creation practice in the development of non-pharmacological interventions for people with Chronic Obstructive Pulmonary Disease: A health CASCADE study | Review |
| 374 | Living in a rural area with advanced chronic respiratory illness: a qualitative study | No data on ethnicity provided |
| 375 | Perceptions of patients about Pulmonary Rehabilitation (PR)in Bangladesh | Conference abstract |
| 376 | Participants perspectives of pulmonary rehabilitation: The role of peer support | Conference abstract |
| 377 | Providing reviews of evidence to COPD patients: qualitative study of barriers and facilitating factors to patient-mediated practice change | No data on ethnicity provided |
| 378 | Chronic obstructive pulmonary disease: Adherence difficulties and suggestions from patients with home oxygen therapy | Conference abstract |
| 379 | Management and Point-of-Care for Tobacco Dependence (PROMPT): a feasibility mixed methods community-based participatory action research project in Ottawa, Canada | Irrelevant |
| 380 | Illness and social factors influence attrition in pulmonary rehabilitation | Conference abstract |
| 381 | The combination of a smoking cessation programme with rehabilitation increases stop-smoking rate | Irrelevant |
| 382 | Physician knowledge and perception of COPD management in Korea and Japan: Continuing to confront COPD (C2C) physician survey 2012-2013 | Conference abstract |
| 384 | Mobile health intervention and COVID-19 pandemic outbreak: insights from Indian context | Irrelevant |
| 385 | Effect of counselling during pulmonary rehabilitation on self-determined motivation to be physically active for people with chronic obstructive pulmonary disease: a pragmatic RCT | RCT |
| 386 | MHealth guideline training for non-communicable diseases in primary care facilities in Nigeria: a mixed methods pilot study | Irrelevant |
| 387 | Prevalence, patterns, and determinants of electronic cigarette and heated tobacco product use in Greece: A cross-sectional survey | Irrelevant |
| 388 | The patient perspective on challenges to participating in pulmonary rehabilitation: An international web-based survey | Conference abstract |
| 389 | Why Do Patients with COPD Decline Rehabilitation | Review |
| 390 | Factors affecting the dietary intake of people on home oxygen therapy (HOT) due to chronic obstructive pulmonary disease | Conference abstract |
| 391 | Factors affecting patients compliance with ambulatory oxygen therapy | Conference abstract |
| 392 | Virtual pulmonary rehabilitation programme - A new era of working | Conference abstract |
| 393 | Improving Uptake of Pulmonary Rehabilitation after a Chronic Obstructive Pulmonary Disease Exacerbation | Conference abstract |
| 394 | Association of culturally competent care with influenza vaccination coverage in the United States | Quantitative |
| 395 | A model of care for patients with COPD in greater cincinnati | Conference abstract |
| 396 | Adapting telehealth pulmonary rehabilitation program to meet the needs of hispanic and African-American patients from disparity communities: A community-based participatory research approach | Conference abstract |
| 397 | Development of Culturally Appropriate Pulmonary Rehabilitation (PR)for Sri Lanka: A qualitative study | Conference abstract |
| 398 | Group-based social identity intervention during pulmonary rehabilitation improves COPD patients experience and promotes compliance with exercise programme | Conference abstract |
| 399 | Lung health of opiate users (LHOP): A pilot study to assess the respiratory health of opiate misusers attending a community substance misuse clinic | Conference abstract |
| 400 | The potential of digital technologies in addressing social isolation experienced with chronic obstructive pulmonary disease (COPD) | Conference abstract |
| 401 | From face-to-face to telerehabilitation: patients prefer a mixed model on a pandemic-free future | Conference abstract |
| 402 | Response shift in COPD patients undertaking pulmonary rehabilitation | Conference abstract |
| 403 | Experiences of training-adherence in a 12 weeks home-based IMT program for individuals with COPD | Conference abstract |
| 404 | Why do patients decline to take part in a research project involving pulmonary rehabilitation? | Irrelevant |
| 405 | Specifications and feasibility of technology-based self-management of COPD: An exploratory qualitative study with patients and providers | Irrelevant |
| 406 | Patients with COPD: Exploring patients' coping ability during an interdisciplinary pulmonary rehabilitation programme: A qualitative focus group study | No data on ethnicity provided |
| 407 | Perceptions of pulmonary rehabilitation by health professionals and culturally and linguistically diverse COPD patients | Conference abstract |
| 408 | Patient-facing Technology for Identification of COPD in Primary Care | Irrelevant |
| 409 | Identifying barriers to smoking cessation in rheumatoid arthritis | Irrelevant |
| 410 | Continuing to confront COPD international physician survey | Conference abstract |
| 411 | Barriers and successful strategies in fostering medication-taking among COPD patients | Conference abstract |
| 412 | Using Experience-Based Co-design to Develop mHealth App for Digital Pulmonary Rehabilitation Management of Patients with Chronic Obstructive Pulmonary Disease (COPD) | Conference abstract |
| 413 | Establishing a pulmonary rehabilitation programme in primary care in Greece: A FRESH AIR implementation study | Irrelevant |
| 414 | Care Coordination for Veterans With COPD: A Positive Deviance Study | Irrelevant |
| 415 | The diverse impact of advance care planning: a long-term follow-up study on patients' and relatives' experiences | Irrelevant |
| 416 | Activating primary care COPD patients with multi-morbidity (APCOM) pilot project: Study protocol | Protocol |
| 417 | Empowerment of primary care patients with chronic obstructive pulmonary disease (COPD) in the context of multi-morbidity by tailored self-management education in Sydney, Australia | Conference abstract |
| 418 | Review of capacity assessments and recommendations for examining capacity | Review |
| 419 | Development and validation of non-adherence to pulmonary rehabilitation questionnaire: A clinical tool for patients with chronic obstructive pulmonary diseases | Irrelevant |
| 420 | Integrating care between an NHS hospital, a community provider and the role of commissioning: The experience of developing an integrated respiratory service | Irrelevant |
| 421 | Determinants of influenza vaccination uptake among Italian healthcare workers | Irrelevant |
| 422 | [A systematic review of shared decision-making interventions for people living with chronic respiratory diseases](https://libsearch.ncl.ac.uk/primo-explore/fulldisplay?docid=RS_609031936Barradell%20Ahronicrespiratorydiseases&context=SP&vid=NEWUI&lang=en_US) | Review |
| 423 | Participant experiences in TANDEM feasibility pilot | Irrelevant |
| 424 | Improving the assessment of medication adherence: Challenges and considerations with a focus on low-resource settings | Irrelevant |
| 425 | Virtual respiratory therapy delivered through a smartphone app: a mixed-methods randomised usability study | Irrelevant |
| 426 | Muscle energy technique for chronic obstructive pulmonary disease: A feasibility study | Irrelevant |
| 427 | Using Electronic Health Record Data to Measure Care Quality for Individuals with Multiple Chronic Medical Conditions | Irrelevant |
| 428 | An interview with Angela Taylor RRT | Irrelevant |
| 429 | Influenza Vaccination among Underserved African-American Older Adults | Irrelevant |
| 430 | Feasibility of completing Multidimensional Dyspnea Profile and Dyspnea-12 over the telephone in patients with oxygen-dependent disease | Irrelevant |
| 431 | Defining Patient-Centered Characteristics of a Telerehabilitation System for Patients with COPD | Conference abstract |
| 432 | The Use of a Smartphone App and an Activity Tracker to Promote Physical Activity in the Management of Chronic Obstructive Pulmonary Disease: Randomized Controlled Feasibility Study | Irrelevant |
| 433 | Implementation of physical activity programs after COPD hospitalizations: Lessons from a randomized study | Irrelevant |
| 434 | Development and Feasibility of a Home Pulmonary Rehabilitation Program With Health Coaching | No data on ethnicity provided |
| 435 | Exercise rehabilitation in telemedicine for COVID-19 patients | Irrelevant |
| 436 | Telemonitoring in chronic ventilatory failure: A new model of survellaince, a pilot study | Irrelevant |
| 437 | Factors associated with medication adherence among people living with COPD: Pharmacists' perspectives | Population not relevant (HCPs) |
| 438 | Protocol for a single-centre mixed-method pre-post single-arm feasibility trial of a culturally appropriate 6-week pulmonary rehabilitation programme among adults with functionally limiting chronic respiratory diseases in Malawi | Protocol |
| 439 | "I only smoke when I have nothing to do": A qualitative study on how smoking is part of everyday life in a Greenlandic village | Irrelevant |
| 440 | Exploring the variation in implementation of a COPD disease management programme and its impact on health outcomes: A post hoc analysis of the RECODE cluster randomised trial | Irrelevant |
| 441 | Self-management programme of activity coping and education-SPACE for COPD(C)-in primary care: a pragmatic randomised trial | RCT |
| 442 | The impact of sport on the physical, psychological and social wellbeing of people with chronic breathlessness: A mixed-methods systematic review | Review |
| 443 | Patients and families realising their future with chronic obstructive pulmonary disease-A qualitative study | No data on ethnicity provided |
| 444 | Improving Exercise-Based Interventions for People Living with Both COPD and Frailty: A Realist Review | Review |
| 445 | Chronic disease patients' experiences with accessing health care in rural and remote areas: a systematic review and qualitative meta-synthesis | Review |
| 446 | Comprehensive pulmonary rehabilitation in home-based online groups: A mixed method pilot study in COPD | No data on ethnicity provided |
| 447 | End-of-Life Discussion, Patient Understanding and Determinants of Preferences in Very Severe COPD Patients: A Multicentric Study | Irrelevant |
| 448 | [La cessazione del fumo in pazienti con malattie respiratorie: Alta priorita, componente integrante della terapia](https://libsearch.ncl.ac.uk/primo-explore/fulldisplay?docid=RS_6182869515nteintegrantedellaterapia&context=SP&vid=NEWUI&lang=en_US) | Not published in English |
| 449 | Are patient preferences for life-sustaining treatment really a barrier to hospice enrollment for older adults with serious illness? | Irrelevant |
| 450 | The effectiveness of a structured education pulmonary rehabilitation programme for improving the health status of people with moderate and severe chronic obstructive pulmonary disease in primary care: The PRINCE cluster randomised trial | RCT |
| 451 | Feasibility, tolerance and effects of adding impact loading exercise to pulmonary rehabilitation in people with chronic obstructive pulmonary disease: study protocol for a pilot randomised controlled trial | Protocol |
| 452 | Augmented reality glasses as a new tele-rehabilitation tool for home use: patients' perception and expectations | Irrelevant |
| 453 | Chronic obstructive pulmonary disease and engagement in occupation | Irrelevant |
| 454 | The efficacy of a flipping education program on improving self-management in patients with chronic obstructive pulmonary disease: a randomized controlled trial | RCT |
| 455 | The evaluation of an interactive web-based Pulmonary Rehabilitation programme: Protocol for the WEB SPACE for COPD feasibility study | Protocol |
| 456 | Integrating patients with chronic respiratory disease and heart failure into a combined breathlessness rehabilitation programme: A service redesign and pilot evaluation | Irrelevant |
| 457 | Experiences of a home-based fall prevention exercise program among older adults with chronic lung disease | Irrelevant |
| 458 | A behaviour change intervention to reduce sedentary time in people with chronic obstructive pulmonary disease: protocol for a randomised controlled trial | Protocol |
| 459 | Determinants of influenza vaccination among the adult and older Italian population with chronic obstructive pulmonary disease A secondary analysis of the multipurpose ISTAT survey on health and health care use | Quantitative |
| 460 | A home-based lower limb-specific resistance training programme for patients with copd: An explorative feasibility study | No data on ethnicity provided |
| 461 | A model for improving obstructive lung disease care for aboriginal australians living in remote Australia | Conference abstract |
| 462 | Oxygen in interstitial lung diseases | Irrelevant |
| 463 | Evaluation of a community pharmacy spirometry testing service for current and recent ex-smokers | Irrelevant |
| 464 | The feasibility of early pulmonary rehabilitation and activity after COPD exacerbations: External pilot randomised controlled trial, qualitative case study and exploratory economic evaluation | No data on ethnicity provided |
| 465 | Home-based pulmonary rehabilitation early after hospitalisation in COPD (early HomeBase): Protocol for a randomised controlled trial | Protocol |
| 466 | Pulmonary rehabilitation referral and participation are commonly influenced by environment, knowledge, and beliefs about consequences: a systematic review using the Theoretical Domains Framework | Review |
| 467 | Impact of feedback on physical activity levels of individuals with chronic obstructive pulmonary disease during pulmonary rehabilitation: A feasibility study | Irrelevant |
| 468 | Long-term oxygen therapy: Review from the patientsĝ perspective | Review |
| 469 | Understanding interprofessional decision-making processes and perceptions of oxygenation for acute respiratory failure | Irrelevant |
| 470 | Perceptions of hyperoxemia and conservative oxygen therapy in the management of acute respiratory failure | Irrelevant |
| 471 | Tasmanian Aborigines step up to health: Evaluation of a cardiopulmonary rehabilitation and secondary prevention program | Irrelevant |
| 472 | Effects of a Community-Based, Post-Rehabilitation Exercise Program in COPD: Protocol for a Randomized Controlled Trial With Embedded Process Evaluation | Protocol |
| 473 | Can electronic monitoring with a digital smart spacer support personalised medication adherence and inhaler technique education in patients with asthma?: Protocol of the randomised controlled OUTERSPACE trial | Protocol |
| 474 | Finding your breath: A novel exercise program for COPD patients | Conference abstract |
| 475 | An innovative COPD early detection programme in general practice: Evaluating barriers to implementation | Irrelevant |
| 476 | Older Patients' Perspectives of Online Health Approaches in Chronic Obstructive Pulmonary Disease | Irrelevant |
| 477 | Cognitive screening in chronic obstructive pulmonary disease: patient's perspectives | Irrelevant |
| 478 | Eleven-month longitudinal study of antibodies in SARS-CoV-2 exposed and naïve primary health care workers upon COVID-19 vaccination | Irrelevant |
| 479 | Understanding end-user perspectives of mobile pulmonary rehabilitation (mPR): Cross-sectional survey and interviews | Irrelevant |
| 480 | Additional evidence for the long-term benefits of pulmonary rehabilitation | Quantitative |
| 481 | Stakeholder-Engaged Derivation of Patient-Informed Value Elements | Irrelevant |
| 482 | Managing mood disorders in patients attending pulmonary rehabilitation clinics | Irrelevant |
| 483 | Developing an intervention to increase REferral and uptake TO pulmonary REhabilitation in primary care in patients with chronic obstructive pulmonary disease (the REsTORE study): mixed methods study protocol | Protocol |
| 484 | Influenza vaccination coverage rates and other related factors in high-risk groups in Birjand, East of Iran | Irrelevant |
| 485 | The last year of life of COPD: a qualitative study of symptoms and services | Irrelevant |
| 486 | The Psychological Impact of Living with Chronic Breathlessness and Experiences of Identification and Assessment of this Symptom in an Older, Frail Population in Primary Care | Irrelevant |
| 487 | Case-finding for COPD in primary care: a qualitative study of patients' perspectives | No data on ethnicity provided |
| 488 | Influenza and pneumococcal vaccination uptake in adults aged ≥65 years and high risk groups admitted to yozgat bozok university research and application hospital | Irrelevant |
| 489 | How does uncertainty shape patient experience in advanced illness? A secondary analysis of qualitative data | Irrelevant |
| 490 | Protocol for a feasibility randomized trial of self-management support for people with chronic obstructive pulmonary disease using lay health coaches | Protocol |
| 491 | Health literacy and medication adherence in older asthmatics | Irrelevant |
| 492 | The role of comorbidities in patients' hypertension self-management | Irrelevant |
| 493 | Older persons' preferences for site of treatment in acute illness | Irrelevant |
| 494 | Mixed-Methods Assessment of a Virtual Reality-Based System for Pulmonary Rehabilitation | No data on ethnicity provided |
| 495 | [Effect of high-deductible health plans on healthcare access, financial strain, medication adherence, and outcomes for patients with COPD: Findings from the national health interview survey](https://libsearch.ncl.ac.uk/primo-explore/fulldisplay?docid=RS_61073449Gaffney%20AWonalhealthinterviewsurvey&context=SP&vid=NEWUI&lang=en_US) | Conference abstract |
| 496 | Barriers to referral to pulmonary rehabilitation in COPD patients from the perspective of general practitioners | Population not relevant (HCPs) |
| 497 | Enlightening chronic obstructive pulmonary disease through patients' and caregivers' narratives | Irrelevant |
| 498 | Adherence to disease management programs in patients with COPD | Irrelevant |
| 499 | Development and validation of the Beliefs and Behaviour Questionnaire (BBQ) | Irrelevant |
| 500 | Persuasive communication in medical decision-making during consultations with patients with limited health literacy in hospital-based palliative care | Irrelevant |
| 501 | Chronic respiratory symptoms and associated factors among cement factory workers in Dejen town, Amhara regional state, Ethiopia, 2015 | Irrelevant |
| 502 | Prevalence of obstructive lung disease (OLD) in persons who inject drugs | Irrelevant |
| 503 | Promoting chronic disease management in persons with complex social needs: A qualitative descriptive study | Irrelevant |
| 504 | The meanings of smoking to women and their implications for cessation | Irrelevant |
| 505 | Self-management for breathlessness in COPD: The role of pulmonary rehabilitation | No data on ethnicity provided |
| 506 | Survey to determine perspectives on the use of spirometry in COPD: Users versus non-users | Irrelevant |
| 507 | Other Ways of Knowing: Considerations for Information Communication in Decision Aid Design | Irrelevant |
| 508 | Implementing telemonitoring in primary care: learning from a large qualitative dataset gathered during a series of studies | Irrelevant |
| 509 | Patient-perceived treatment burden of chronic obstructive pulmonary disease | No quotations from minority ethnic groups |
| 510 | Integrating psychological screening into reviews of patients with COPD | Irrelevant |
| 511 | Factors affecting the offer of pulmonary rehabilitation | Population not relevant (HCPs) |
| 512 | Dance for people with chronic breathlessness: A transdisciplinary approach to intervention development | Irrelevant |
| 513 | 'Consumed by breathing' - A critical interpretive meta-synthesis of the qualitative literature | Review |
| 514 | Minimizing the evidence-practice gap - a prospective cohort study incorporating balance training into pulmonary rehabilitation for individuals with chronic obstructive pulmonary disease | Irrelevant |
| 515 | Perspectives of healthcare professionals and patients on the application of mindfulness in individuals with chronic obstructive pulmonary disease | Population not relevant (HCPs) |
| 516 | Psychological considerations in pulmonary rehabilitation | Irrelevant |
| 517 | Self-efficacy for physical activity and insight into its benefits are modifiable factors associated with physical activity in people with COPD a mixed-methods study | Irrelevant |
| 518 | Development and Content Validation of a Comprehensive Health Literacy Survey Instrument for Use in Individuals with Asthma during the COVID-19 Pandemic | Irrelevant |
| 519 | Understanding the experience of patients with chronic obstructive pulmonary disease who access specialist palliative care: a qualitative study | Irrelevant |
| 520 | Measuring the value of integrated respiratory consultant support of a community respiratory multidisciplinary team (MDT) | Irrelevant |
| 521 | What are the perceived influences on asthma self-management at the workplace? A qualitative study | Irrelevant |
| 522 | Smoking cessation counselling in general practice for COPD smokers: Determinants for general practitioners' compliance with a treatment protocol | Population not relevant (HCPs) |
| 523 | Daily utility and satisfaction with rollators among persons with chronic obstructive pulmonary disease | Irrelevant |
| 524 | Barriers and facilitators influencing self-management among COPD patients: a mixed methods exploration in primary and affiliated specialist care | Irrelevant |
| 525 | Understanding reasons for nonadherence to medications in a medicare Part D beneficiary sample | Irrelevant |
| 526 | Dietary supplementation by Japanese patients with chronic obstructive pulmonary disease | Irrelevant |
| 527 | Feasibility and acceptability of introducing advance care planning on a thoracic medicine inpatient ward: An exploratory mixed method study | Irrelevant |
| 528 | Adherence and factors affecting satisfaction in long-term telerehabilitation for patients with chronic obstructive pulmonary disease: A mixed methods study eHealth/ telehealth/ mobile health systems | No data on ethnicity provided |
| 529 | Pulmonary rehabilitation for interstitial lung disease: Referral and patient experiences | Irrelevant |
| 530 | Benefits and costs of home-based pulmonary rehabilitation in chronic obstructive pulmonary disease - a multi-centre randomised controlled equivalence trial | RCT |
| 531 | Exploring the Views of Individuals With Chronic Obstructive Pulmonary Disease on the Use of Rollators: A QUALITATIVE STUDY | Irrelevant |
| 532 | Comparison of a structured home-based rehabilitation programme with conventional supervised pulmonary rehabilitation: a randomised non-inferiority trial | Irrelevant |
| 533 | Healthcare access and disparities in chronic medical conditions in Urban populations | Irrelevant |
| 534 | Web-Based Self-management Program (SPACE for COPD) for Individuals Hospitalized With an Acute Exacerbation of Chronic Obstructive Pulmonary Disease: Nonrandomized Feasibility Trial of Acceptability | Irrelevant |
| 535 | Impact of a pharmacist-led outpatient telemedicine clinic on chronic obstructive pulmonary disease in a veteran population | Irrelevant |
| 536 | Machine learning to identify and understand key factors for provider-patient discussions about smoking | Irrelevant |
| 537 | The patient perspective; knowledge and memory influence pulmonary rehabilitation referral | Conference abstract |
| 538 | Implementation and evaluation of a physical activity counselling programme in primary care among cancer survivors: SoDA study protocol | Protocol |
| 539 | Receipt of Primary care linked to high-value care, better health care experience | Irrelevant |
| 540 | Tele-rehabilitation for patients who have been hospitalised with Covid-19: a mixed-methods feasibility trial protocol | Protocol |
| 541 | Article experience of patients with COPD of pharmacists' provided care: A qualitative study | Irrelevant |
| 542 | Experience of Patients with COPD of Pharmacists' Provided Care: A Qualitative Stud | Irrelevant |
| 543 | Determinants of frailty in primary care patients with COPD: the Greek UNLOCK study | Irrelevant |
| 544 | Adherence to inhalers and comorbidities in COPD patients. A cross-sectional primary care study from Greece | Quantitative |
| 545 | Development of an Electronic Interdisciplinary Chronic Obstructive Pulmonary Disease (COPD) Proforma (E-ICP) to Improve Interdisciplinary Guideline Adherence in the Emergency Department: Modified Delphi Study | Irrelevant |
| 546 | Mapping of Modifiable Factors with Interdisciplinary Chronic Obstructive Pulmonary Disease (COPD) Guidelines Adherence to the Theoretical Domains Framework: A Systematic Review | Review |
| 547 | A Qualitative Study of Pulmonary and Palliative Care Clinician Perspectives on Early Palliative Care in Chronic Obstructive Pulmonary Disease | Irrelevant |
| 548 | Recommendations following a modified UK-Delphi consensus study on best practice for referral and management of severe asthma | Irrelevant |
| 549 | How resources determine pulmonary rehabilitation programs: A survey among Belgian chest physicians | Population not relevant (HCPs) |
| 550 | Protocol for the cultural adaptation of pulmonary rehabilitation and subsequent testing in a randomised controlled feasibility trial for adults with chronic obstructive pulmonary disease in Sri Lanka | Irrelevant |
| 551 | Vaccination and modern management of chronic obstructive pulmonary disease-a narrative review | Review |
| 552 | Implementation of COPD guidelines is low for pulmonary rehabilitation and exacerbation plans: Pilot data | Irrelevant |
| 553 | Why are some evidence-based care recommendations in chronic obstructive pulmonary disease better implemented than others? perspectives of medical practitioners | Irrelevant |
| 554 | Barriers to, and facilitators for, referral to pulmonary rehabilitation in COPD patients from the perspective of Australian general practitioners: a qualitative study | Population not relevant (HCPs) |
| 555 | Protocol for a feasibility trial to inform the development of a breathlessness rehabilitation programme for chronic obstructive pulmonary disease and chronic heart failure (the COHERE trial) | Protocol |
| 556 | Supported self-management for patients with moderate to severe chronic obstructive pulmonary disease (COPD): An evidence synthesis and economic analysis | Irrelevant |
| 557 | Assessment of Physical Health Status, Depression and Activities of Daily Living Among Chronic Obstructive Pulmonary Disease Patients in Tertiary Care Hospitals in Mangalore, India | Irrelevant |
| 558 | A virtual reality–supported intervention for pulmonary rehabilitation of patients with chronic obstructive pulmonary disease: Mixed methods study | No data on ethnicity provided |
| 559 | Fit 4 surgery, a bespoke app with biofeedback delivers rehabilitation at home before and after elective lung resection | Irrelevant |
| 560 | Shared decision making and experiences of patients with long-term conditions: has anything changed? | Irrelevant |
| 561 | Qualitative investigation into a wearable system for chronic obstructive pulmonary disease: The stakeholders' perspective | Irrelevant |
| 562 | What prevents people with chronic obstructive pulmonary disease from attending pulmonary rehabilitation? A systematic review | Review |
| 563 | Living with chronic lung disease: An occupational perspective | Irrelevant |
| 564 | Cardiovascular diseases preventive policy-making process in Iran: A framework-based policy analysis | Irrelevant |
| 565 | Adherence in the case of chronic diseases: patient-centred approach is needed | Conference abstract |
| 566 | Reflexive thematic analysis exploring stakeholder experiences of virtual pulmonary rehabilitation (VIPAR) | Population not relevant (HCPs) |
| 567 | Patients’ experiences with participating in a team-based person-centred intervention for patients at risk of or diagnosed with COPD in general practice | Irrelevant |
| 568 | Self-management behaviors to reduce exacerbation impact in COPD patients: A delphi studySelf-management behaviors to reduce exacerbation impact in COPD patients: A delphi study | Irrelevant |
| 569 | Strategies to Improve Enrollment and Participation in Pulmonary Rehabilitation Following a Hospitalization for COPD RESULTS OF A NATIONAL SURVEY | Irrelevant |
| 570 | Efficacy of confrontational counselling for smoking cessation in smokers with previously undiagnosed mild to moderate airflow limitation: Study protocol of a randomized controlled trial | Protocol |
| 571 | The Economic Burden of Chronic Obstructive Pulmonary Disease in Greece | Irrelevant |
| 572 | COVID-19 vaccination hesitancy and uptake: Perspectives from people released from the Federal Bureau of Prisons | Irrelevant |
| 573 | Financial Hardship From Medical Bills Among Adults With Chronic Liver Diseases: National Estimates From the United States | Irrelevant |
| 574 | How do healthcare professionals perceive physical activity prescription for community-dwelling people with COPD in Australia? A qualitative study | Irrelevant |
| 575 | Evaluation of mhealth intervention in copd: Caremessage | Conference abstract |
| 576 | A motivational intervention for patients with COPD in primary care: qualitative evaluation of a new practitioner role | Irrelevant |
| 577 | Targeted prevention in primary care aimed at lifestyle-related diseases: a study protocol for a non-randomised pilot study | Protocol |
| 578 | How do general practitioners implement decision-making regarding COPD patients with exacerbations? An international focus group study | Irrelevant |
| 579 | Patient engagement in interprofessional team-based chronic disease management: A qualitative description of a Canadian program | Irrelevant |
| 580 | The role of telemedicine | Irrelevant |
| 581 | Long-term quit rates after a perioperative smoking cessation randomized controlled trial | RCT |
| 582 | A description of the management of patients with COPD in the primary care setting of Antwerp | Conference abstract |
| 583 | Development of a self-administered questionnaire to identify levers and barrier of adhesion behavior to patient's medication: Quilam | Irrelevant |
| 584 | The long-term outcomes of tobacco control strategies based on the cognitive intervention for smoking cessation in COPD patients | Irrelevant |
| 585 | Fidelity and Feasibility of a Brief Emergency Department Intervention to Empower Adults With Serious Illness to Initiate Advance Care Planning Conversations | Irrelevant |
| 586 | User experiences and perceived effectiveness of an eHealth self-management intervention in COPD patients with heart failure | Irrelevant |
| 587 | An intervention for pulmonary rehabilitators to develop a social identity for patients attending exercise rehabilitation: A feasibility and pilot randomised control trial protocol | Protocol |
| 588 | Improving physical healthcare for people who use heroin and crack cocaine | Irrelevant |
| 589 | Tai Chi Movements for Wellbeing – evaluation of a British Lung Foundation pilot | Irrelevant |
| 590 | Impact of an Animation Education Program on Promoting Compliance With Active Respiratory Rehabilitation in Postsurgical Lung Cancer Patients A Randomized Clinical Trial | RCT |
| 591 | Telehomecare for patients with multiple chronic illnesses: Pilot study | Irrelevant |
| 592 | Understanding influences on the uptake of pulmonary rehabilitation in the East of England: An inclusive design/mixed-methods study protocol | Protocol |
| 593 | Understanding the influences of copd patient’s capability on the uptake of pulmonary rehabilitation in the uk through an inclusive design approach | No data on ethnicity provided |
| 594 | Addressing pressures on health services in Belo Horizonte, Brazil through community-based care for poor older people: a qualitative study | Irrelevant |
| 595 | Achieving therapeutic clarity in assisted personal body care: professional challenges in interactions with severely ill COPD patients | Irrelevant |
| 596 | Improving Quality of Life in Chronic Obstructive Pulmonary Disease by Integrating Palliative Approaches to Dyspnea, Anxiety, and Depression | Irrelevant |
| 597 | Expanding pharmacists’ roles: Pharmacists’ perspectives on barriers and facilitators to collaborative practice | Irrelevant |
| 598 | Pan-Canadian asthma and COPD standards for electronic health records: A Canadian Thoracic Society Expert Working Group Report | Irrelevant |
| 599 | What influenced people with chronic or refractory breathlessness and advanced disease to take part and remain in a drug trial? A qualitative study | Irrelevant |
| 600 | Maintenance of non-pharmacological strategies 6 months after patients with chronic obstructive pulmonary disease (COPD) attend a breathlessness service: A qualitative study | Irrelevant |
| 601 | Managing respiratory disease: The role of a psychologist within the multidisciplinary team | Irrelevant |
| 602 | Singing for People with Advance Chronic Respiratory Diseases: A Qualitative Meta-Synthesis | Review |
| 603 | The impact of community-based pulmonary rehabilitation on the health and lives of migrant workers with pneumoconiosis in China: a qualitative study exploring patient experience | Irrelevant |
| 604 | Primary care oncology model (PCOM): Implementation of a model integrating primary and oncology care for patients taking oral anticancer agents | Irrelevant |
| 605 | Medication adherence in the older adults with chronic multimorbidity: a systematic review of qualitative studies on patient’s experience | Review |
| 606 | Adherence to Exercise Training in COPD Patients- Factors that Predict Patient Adherence and Non-Adherence- A Preliminary Study | No data on ethnicity provided |
| 607 | Understanding factors that inhibit or promote the utilization of telecare in chronic lung disease | Irrelevant |
| 608 | MBCT for Patients with Respiratory Conditions Who Experience Anxiety and Depression: A Qualitative Study | Irrelevant |
| 609 | Pulmonary rehabilitation and severe exacerbations of COPD: Solution or white elephant? | Irrelevant |
| 610 | A telepharmacy intervention to improve inhaler adherence in veterans with chronic obstructive pulmonary disease | Irrelevant |
| 611 | Strategies to measure and improve patient adherence in clinical trials | Irrelevant |
| 612 | “It is the fear of exercise that stops me” – attitudes and dimensions influencing physical activity in pulmonary hypertension patients | Irrelevant |
| 613 | 'All illness is personal to that individual': A qualitative study of patients' perspectives on treatment adherence in bronchiectasis | Irrelevant |
| 614 | Engaging high-risk groups in early lung cancer diagnosis: a qualitative study of symptom presentation and intervention preferences among the UK's most deprived communities | Irrelevant |
| 614 | Taking charge: A proposed psychological intervention to improve pulmonary rehabilitation outcomes for people with copd | Irrelevant |
| 615 | A mixed methods study of Aboriginal health workers' and exercise physiologists' experiences of co-designing chronic lung disease 'yarning' education resources | Irrelevant |
| 616 | Strengthening the role of the primary health care in the covid-19 response: Evidence from yerevan | Irrelevant |
| 617 | Describing the delivery of clinical pharmacy services via telehealth: A systematic review | Review |
| 618 | Self-assessment of adherence to medication: A case study in campania region community-dwelling population | Irrelevant |
| 619 | [The ancient Tuberculosis in the novel COVID-19 scenario](https://libsearch.ncl.ac.uk/primo-explore/fulldisplay?docid=RS_619848250e20741isinthenovelcovidscenario&context=SP&vid=NEWUI&lang=en_US) | Irrelevant |
| 620 | Adoption of Patient Engagement Strategies by Physician Practices in the United States | Irrelevant |
| 621 | A patient-centred approach to health service delivery: improving health outcomes for people with chronic illness | Irrelevant |
| 622 | General practitioners' perceptions of COPD treatment: thematic analysis of qualitative interviews | Population not relevant (HCPs) |
| 623 | The quality of obstructive lung disease care for adults in the United States as measured by adherence to recommended processes | Irrelevant |
| 624 | Disability-adjusted life years (DALYs) for 291 diseases and injuries in 21 regions, 1990-2010: a systematic analysis for the Global Burden of Disease Study 2010 | Irrelevant |
| 625 | Current developments in behavioral interventions for tobacco cessation | Irrelevant |
| 626 | How patient experience informed the SafeMed Program: Lessons learned during a Health Care Innovation Award to improve care for super-utilizers | Irrelevant |
| 627 | Implementing an Advance Care Planning Intervention in Community Settings with Older Latinos: A Feasibility Study | Irrelevant |
| 628 | Acceptance and practicability of a visual communication tool in smoking cessation counselling: a randomised controlled trial | RCT |
| 629 | Randomized Controlled Trial of an Internet-Based Versus Face-to-Face Dyspnea Self-Management Program for Patients With Chronic Obstructive Pulmonary Disease: Pilot Study | RCT |
| 630 | Lung Association's Ottawa COPD program: A successful maintenance pulmonary rehabilitation program | Irrelevant |
| 631 | Capnography-assisted learned, monitored (CALM) breathing therapy for dysfunctional breathing in COPD: A bridge to pulmonary rehabilitation | Irrelevant |
| 632 | Mind-Body Intervention for Dysfunctional Breathing in Chronic Obstructive Pulmonary Disease: Feasibility Study and Lessons Learned | Irrelevant |
| 633 | The preferences of people with asthma or chronic obstructive pulmonary disease for self-management support: A qualitative descriptive study | Irrelevant |
| 634 | Use of telemedicine in the assessment of patients referred for pulmonary rehabilitation | No data on ethnicity provided |
| 635 | Identifying needs of highly nicotine dependent COPD smokers using a stepped care model of psychological intervention | Conference abstract |
| 636 | Understanding the factors affecting self-management of COPD from the perspectives of healthcare practitioners: a qualitative study | Population not relevant (HCPs) |
| 637 | Findings of the Chronic Obstructive Pulmonary Disease-Sitting and Exacerbations Trial (COPD-SEAT) in Reducing Sedentary Time Using Wearable and Mobile Technologies With Educational Support: Randomized Controlled Feasibility Trial | Irrelevant |
| 638 | LungFlareCare: Development and evaluation of a new web-based educational resource | Irrelevant |
| 639 | Development of a patient-centred, evidence-based and consensus-based discharge care bundle for patients with acute exacerbation of chronic obstructive pulmonary disease | Irrelevant |
