# Supplementary material for: Barriers and facilitators of adherence to treatment interventions for COPD amongst individuals from minority ethnic communities: Meta-ethnography
Source: PLoS One. 2025 Feb 10;20(2):e0318709. doi: 10.1371/journal.pone.0318709 (PMC11809908; doi:10.1371/journal.pone.0318709)
Supplement: S4 Table — (DOCX) [file pone.0318709.s004.docx]

**S3 Table: Result of the qualitative data synthesis**

**Table 1: Theme 1: Positive and negative experiences affecting a person’s motivation for their care**

| **Synthesised themes (third order constructs)** | **Sub-themes** | **Second order constructs: the authors interpretations of the original findings** | **First Order constructs: examples of direct quotations from the participants of the study** |
| --- | --- | --- | --- |
| Positive and negative experiences affecting a person’s motivation for their care | The influence of healthcare professionals | Experiences of interactions with health professionals | *“I had a hospitalization, went to see [the] doctor, and he told me basically I had less than five years to live. See you later. And that’s how it was put to me... We still [had] a few questions for the surgeon, but it turned into “oh, it’s a very busy clinic today.” You know, I’m supposed to trust this man to open me up and take my lung out, and it’s money, it all comes back to money” (Levack et al, 2016)*  *Māori* |
|  |  |  | “All the Paˉkehaˉ drugs, all the – the antibiotics, the prednisone – they were all messing with my weight, messing with my head, just put me in a dark place. And I didn’t want to get out of bed. Didn’t want to exercise. At that stage I just ready to give up. And then God sent me [the marae staff]. And it’s those people that have got me to where I am now.” *(Levack et al, 2016)*  *Māori* |
|  |  |  | *“I didn’t know these groups existed. I didn’t even know [the marae] had a hauora [Maˉori health service]. Until she [the marae nurse] came to my place, or actually it was my partner they’d rung... and they came round. I’m looking – “Who are these two jungle bunnies come here?” And they told me – they read up on what I had you know. I said “Jesus, these people came straight off the street, they know what I got.” So they ended up dragging me along – not dragging me, but getting me to come here, you know.” (Levack et al, 2016)*  *Māori* |
|  |  | Words of commiseration and encouragement from other members | *“I told the doctor that I don’t have the strength and the will power (to quit smoking) and he said*: “*I cannot deal with you*. *If you don’t quit smoking, I will see you with an oxygen tank in one year.” And that made me feel very bad.” (Glasser et al, 2016)*  *Hispanic* |
|  |  | Some participants have had positive experiences in obtaining and managing medications. | *“I don’t have problems (with medications) because I have nurses who come to my house. They come every week on Tuesdays and Fridays. They are lovely, lovely, responsible people*.” *(Glasser et al, 2016)*  *Hispanic* |
|  |  | Past experiences of exercise | *“Was there anything about the class that made you nervous before you started, or when you first started going? The exercise. I used to think “oh,” you know. and the physio will say “no, keep going, keep going,” you know, you think “I can’t breathe.” I used to be afraid of the breathing.” (Levack et al, 2016)*  *Māori* |
|  |  | Past experiences of health services | *“I didn’t like it... They said I had to do this; do that. But they didn’t explain anything about the health, what it does to you... What stopped you coming back?*  *I went to ah, that department, ah, where you blow these things in the tube... I chucked it in too, that one. Pissed me off... Well they told me to blow, blow, blow, blow, keep blowing. and that was – I had a severe – ah, breathing problems! so I chucked it in.” (Levack et al, 2016)*  *Māori* |
|  |  |  | *“Oh, I had um – on one of our courses to do with my diabetes... and I ended up with um, being wheel-chaired, ah, to a&e [accident & emergency]. Cause I had a – my muscles pulled and that, I couldn’t – [it] was like – sciatica. so, went to the doctors, the only thing they said is um, tramadol... [so for pulmonary rehabilitation in the hospital] You’re in the right place. If anything goes down.” (Levack et al, 2016)*  *Māori* |
|  |  | Program experiences | *“I like the atmosphere... and although we do exercise, and oh, we all hate doing them. But we do, we put an effort into them... And I love it up here, I love coming here, it’s just – the atmosphere’s totally different [from hospital], and it’s beautiful.” (Levack et al, 2016)*  *Māori* |
|  |  | Positive experience of pulmonary rehabilitation | *“What I liked and enjoyed most was that it gave me a routine and took me out of the house, I felt good mingling with other people, I enjoyed interacting with them, it helped me relax.” (Early et al, 2020)*  *British Pakistani* |
|  |  |  | *“But everything is already excellent, the exercise coaches are excellent, they provide you with refreshments what else. I found everything excellent.” (Early et al, 2020)*  *British Pakistani* |
|  |  |  | *“InshaAllah (God willing) I would be very interested in attending it the second time, I think it’s important for us to take steps that are good for our health and this is a fantastic opportunity that takes you away from your daily routine, takes you out of the house and allows you to relax. Otherwise, it’s not the same when we go out with the family and then there are other issues that come with it. Going to these exercise classes ties you to a routine which is very good, it commits you to a system which is very helpful.” (Early et al, 2020)*  *British Pakistani* |
|  |  | Receiving advice from doctors | *“I quit smoking because my doctor said to quit. I had shortness of breath and declining lung function” (Poureslami et al, 2015)*  *Chinese in Canada* |
|  |  |  | *“My doctor told me if I don't quit I will die. I quit smoking because of my COPD, I quit the next day”. (Poureslami et al, 2015)*  *Chinese in Canada* |
|  |  |  | *“I know smoking is bad but I don’t know how to quit.” (Poureslami et al, 2015)*  *Chinese in Canada* |
|  |  | Approach, Recruitment and Enrollment | “*Like I said, my doctor never told me anything about that [PR]. I mean, he could tell his patients when they have severe – chronic breathing, respiratory infections, and things like that. He should advise his patients to just give it a try. If he would have told me to go, I would have went”* (Polo et al, 2023)  *Hispanic* |
|  |  | Pulmonary rehabilitation referral | *“Nobody said anything. No, no. I don’t know anything about that.” (Early et al, 2020)*  *British Pakistani* |
|  |  |  | *“No, they have not referred me, but I had spoken to a doctor. He is a very nice doctor. I told him that I have been contacted by this programme people, should I attend or not? He said its a very good thing, do attend.” (Early et al, 2020)*  *British Pakistani* |
|  |  |  | *“No, I have never been referred. They just ask me to continue doing some exercises. I get lot of exercises since I do all the housework. But I have never been told what you are saying.” (Early et al, 2020)*  *British Pakistani* |
|  | Social support from family, friends, and peers | Tensions of balancing support with independence (Mismatch and mistrust) | *“That’s what annoyed me because what I was telling them, they weren’t taking any notice and that really gets on your nerves because you’re the one in pain.” (Brighton et al, 2020)*  *Asian, Black, or Mixed* |
|  |  | Some of the participants were very bothered by second-hand smoke in their environment. | *“I had neighbors who smoked. I stayed on the third floor and they smoked on the steps- it came up to my apartment. There was nothing I could do about it.” (Glasser et al, 2016)*  *African American* |
|  |  | Felt disinclined to attend programs where they might be the only Maˉori person attending. | *“How it was for me? – I felt very left out, I felt like – being a Maˉori, you know. They were all in their own individual group, you know, the Paˉkehaˉ [non-Maˉori New Zealanders]... I stuck out like a sore thumb, being the only Maˉori there.” (Levack et al, 2016)*  *Māori* |
|  |  | Peer support | *“I went to like 3 or 4 classes in a row, which is like 3 or 4 weeks. But then I started getting a little bit um, probably a bit too proud, or thinking about nah these, these people are too old for me, or the other way around, I’m too young.” (Levack et al, 2016)*  *Māori* |
|  |  |  | *“Once you’re in this class, and you hear the korero (Maˉori word for “conversation”) around the table, you do, you get over what you’re feeling, because there’s others behind you that are feeling either worse, or better. And its just – for me, it was sort of like a – a healing part for me.” (Levack et al, 2016)*  *Māori* |
|  |  | Being part of a group | *“Basically, the biggest thing I struggled with was that I was. I’m only 42. And how many people 40, 60, 70, 80 year olds [were at pulmonary rehabilitation]? I struggled.” (Levack et al, 2016)*  *Māori* |
|  |  | Work environment and being forced to smoke by older peers | *“Older school mates forced me to have myf irst smoke.” (Poureslami et al, 2015)*  *Chinese in Canada* |
|  |  |  | *“My relatives gave me cigarettes to smoke when I was young and then I became addicted” (Poureslami et al, 2015)*  *Chinese in Canada* |
|  |  |  | *“I was exposed to 2^nd^ hand smoke at home for 20 years because of my father and husband were heavy smokers, then I started to smoke” (Poureslami et al, 2015)*  *Chinese in Canada* |
|  |  |  | *“I tried to quit smoking [cold turkey] for many times, but I failed because I was still working and surrounding friends were smokers and work industry influenced me along with social interaction. At the end, I quit because my doctor told him I needed to quit.” (Poureslami et al, 2015)*  *Chinese in Canada* |
|  |  | Curiosity and social matters when they were young | *“Because everyone smoked back then, and to look more mature.” (Poureslami et al, 2015)*  *Chinese in Canada* |
|  |  |  | *“When I was young, I was curious and started smoking when I saw other people smoking. I started smoking because of my favorite movie actor” (Poureslami et al, 2015)*  *Chinese in Canada* |
|  |  |  | *“I started smoking because my friends smoked and I thought it was cool and fun” (Poureslami et al, 2015)*  *Chinese in Canada* |
|  |  | Difficulty in maintaining their non-smoking status | *“I quit once, then I started to smoke again because smoking helps me build close relationships with my friends when we play cards” (Poureslami et al, 2015)*  *Chinese in Canada* |
|  |  | Seeing others succeed gives him the confidence that he too can successfully participate in the program. | *“The way [Mr. Lopez] here was doing the bike, when we see the video, we get motivated seeing him doing it...the way he’s breathing is excellent according to what I saw there.”*  *“It got me out of my room with my anxiety and my depression—it motivated me to do other things that I didn’t do before” (Pekmezaris et al, 2020)*  *Hispanic or African American* |
|  |  | Friends and family members were also supporting and aiding cessation | *“After coming to Canada, I was forced to quit smoking by my family, my daughters told me to quit and yelled at me.” (Poureslami et al, 2015)*  *Chinese in Canada* |
|  |  |  | *“My biggest motivation to quit was my older brother – he convinced me to quit.” (Poureslami et al, 2015)*  *Chinese in Canada* |
|  | The impact of underlying health conditions | Health effect | *“Yeah, and my last class I couldn’t do their stair exercise, and it’s only four steps. Yeah, and that, that, that disheartens me as well. You know, I can’t even do four steps, and then you watch um, the elderly lady going up. It makes me angry that I’ve got myself in that position. Yeah. But yeah, I know that that’s my only way out, is to actually go to those classes.” (Levack et al, 2016)*  *Māori* |
|  |  |  | *“[To start with] I just thought “oh, what we’re going – going to get out of it?” But I thought I’ll keep going. But then I said to them, when I’d been to it for a few times, I used to say, “oh, I must admit, it has been doing me good.” I said – before I used to think oh exercise was just a load of – it’s not going to help you, you know, with all the breathlessness and all that. It’s not going to help you.” (Levack et al, 2016)*  *Māori* |
|  |  | Past experiences of having COPD | *“And that exercise in the hospital gym was rubbish. We had to be there at nine o’clock in the morning, I mean anyone with COPD or severe emphysema know, that’s not a really for us. It takes me over an hour just to have a shower.” (Levack et al, 2016)*  *Māori* |
|  |  | Health reasons (e.g., constant coughing and short of breath) | *“[After learning about health issues of smoking] I wanted to avoid exposure to smoke. I quit my job at the Casino.” (Poureslami et al, 2015)*  *Chinese in Canada* |
|  |  |  | *“When you quit smoking, your lung function improves, my doctor said.” (Poureslami et al, 2015)*  *Chinese in Canada* |
|  |  |  | *“Smoking makes the body weaker and you get sick more often” (Poureslami et al, 2015)*  *Chinese in Canada* |
|  |  | Disease severity (having COPD) | *“I cut down smoking because I hospitalized for my lung problem, then I cut my one-pack a day smoking [sic] to three packs a month. “(Poureslami et al, 2015)*  *Chinese in Canada* |
|  |  |  | *“I quit because I had a stroke and was admitted to hospital for 3 weeks’’ (Poureslami et al, 2015)*  *Chinese in Canada* |
|  |  |  | *“When I quit my COPD was around the same so I continue again” (Poureslami et al, 2015)*  *Chinese in Canada* |
|  |  | Success in quitting smoking | *“I know bad health well. I was in intensive care and I believed that I was going to die. When you see this you quit cigarettes. You say, “Oh my God, if you continue this way you are going to die.” (*Glasser *et al,* 2016)  *Hispanic* |
|  | Restrictions and regulations in the place they live | Immigration to Canada and restrictions on smoking in the public | *“Smoking is harder here (I had to smoke outside) and I didn't want to smoke in front of the children. When it is too cold I do not go out to smoke, so I quit.” (Poureslami et al, 2015)*  *Chinese in Canada* |
|  |  |  | *“I quit smoking after coming to Canada and observed that it’s not culturally good habit to smoke.” (Poureslami et al, 2015)*  *Chinese in Canada* |
|  |  | Cost | *“Smoking is expensive.” (Poureslami et al, 2015) –*  *Chinese in Canada* |

**Table 2: Theme 2: Patients’ attitudes and beliefs**

| **Synthesised themes (third order constructs)** | **Sub-themes** | **Second order constructs: the authors interpretations of the original findings** | **First Order constructs: examples of direct quotations from the participants of the study** |
| --- | --- | --- | --- |
| Patients’ attitudes and beliefs | Looking to improve their own health | Seeking a change | *“I’ll go and try anything, I’ve done that a lot, I thought I’ll get there somehow but do something positive. As long as I’m doing something positive to help myself, if you like, I’ll do it.” (Brighton et al, 2020)*  *Asian, Black, or Mixed* |
|  |  | Health control | *“I liked them a lot, I wanted to see if this worked for me. It wasn’t easy at first because I wasn’t used to it, but now I can even do them on my own.” (Pekmezaris* *et al, 2020)*  Hispanic or African American |
|  |  |  | *“It was hard in the beginning. Because when you don’ t exercise for a long time and then you start—it is stressful but then gets easier. I was always allowed to rest during the sessions. I felt an 80% improvement. My chest isn’t that tight and I can breathe through my nose when I do the breathing exercises that I learned in the program.” (Pekmezaris* *et al, 2020)*  Hispanic or African American |
|  | Personal beliefs and drive | Attitudes were often attributed to personal strengths or self-perceived flaws | *“I’m just too proud to do stuff like that [catch the free shuttle to a hospital-based program]” (Levack et al, 2016)*  *Maˉori* |
|  |  |  | *“I used to have a bad attitude. I used to think, oh, exercise ain’t going to help me, you know... At first I really had a bit of a negative attitude to it. Cause I have negative attitude to everything. and I’m like “nah, I don’t want to go to that.” You know, I lose interest in things quick. real quick.” (Levack et al, 2016)*  *Māori* |
|  |  |  | “I mean that’s why I wasn’t there at the end of last year, cause I was still in that dark place, and I was finding excuses every week, why I’m not coming. even though [the community nurse] and them are trying to push me here, and push me here. Because I wasn’t in the right head space for myself, I didn’t want to bring anyone else down.” *(Levack et al, 2016)*  *Māori* |
|  |  |  | *“Nobody else can help me but myself, so I thought it was time to kick myself in the butt, because okay, I weighed in at a hundred and twenty-six (kilograms).” (Levack et al, 2016)*  *Māori* |
|  | Placing trust in healthcare providers | South Asian patients placed high value on following healthcare advice | *“I don’t remember accurately but I remember that I used to go once a week so in total I went about 2-3 times but I do remember feeling good after the exercise. But then exercise is good for health. But I try to do it at home whenever I remember because I get easily distracted with the house chores so I do try to do it as often as I can. But I would say if your doctor recommends then one should listen to the doctor and attend these classes because it is for our own benefit, no one else benefits from it apart from one own self. Exercise helps to make our lungs stronger.” (Early et al, 2020)*  *British Pakistani* |
|  |  |  | *“I do want to attend. They will tell me useful things.” (Early et al, 2020)*  *British Pakistani* |
|  |  |  | *“No they did not tell me anything at all [about what happens at PR]. They said it’s a very good thing, do go. They said will you go if we refer? I said yes.” (Early et al, 2020)*  *British Pakistani* |
|  |  |  | *“Yes I will be interested. I will definitely go if they ask me to.” (Early et al, 2020)*  *British Pakistani* |
|  |  |  | *“I don’t think anybody would not want to go. I would always want to attend such classes that will benefit me.” (Early et al, 2020)*  *British Pakistani* |
|  |  |  | *“[When the hospital referred me] they did not explain much as such but they did ask me if I wanted to go the exercise classes, So I said OK I will go then they prepared a letter.” (Early et al, 2020)*  *British Pakistani* |
|  |  |  | *“I think one should prioritize one’s health and not think about the differences for example this person is a white or this person is an Indian or Pakistani, because this is a support and help being provided for us, it is for us and the services are free they were created to help us get better. I think we should all support the services.” (Early et al, 2020)*  *British Pakistani* |
|  | Patients’ perception of smoking and pulmonary rehabilitation program |  |  |
|  |  | Reluctant to quit because they thought it was too late | “The time already passed by - It’s hard to change my habit because I’m already old and have the disease [COPD].” *(Poureslami et al, 2015)*  *Chinese in Canada* |
|  |  | Smoking helped to relax, help in reducing COPD-related anxiety and stress, smoking was also thought to help fight disease/germs and re-energize people | *“Smoking helps with disinfection” (Poureslami et al, 2015)*  *Chinese in Canada* |
|  |  |  | *“Smoking gives you energy/recharge/revitalize and a psychological habit.” (Poureslami et al, 2015)*  *Chinese in Canada* |
|  |  |  | *“Smoking helps during weather change when not feeling well” (Poureslami et al, 2015)*  *Chinese in Canada* |
|  |  |  | *“There is no good replacement for getting focused. Smoking is the best thing to help me relaxed” (Poureslami et al, 2015)*  *Chinese in Canada* |
|  |  |  | *“Nicotine calms me down’. (Poureslami et al, 2015)*  *Chinese in Canada* |
|  |  |  | *‘I felt more relaxed when I smoked.” (Poureslami et al, 2015)*  *Chinese in Canada* |
|  |  |  | *“I smoke 1-1/5 packs a day as smoking triggers my brain to think” (Poureslami et al, 2015)*  *Chinese in Canada* |
|  |  |  | *“My smoking depends on my mood: when I’m happy, then I don’t smoke, when I’m unhappy and with stress [sic] I smoke.” (Poureslami et al, 2015)*  *Chinese in Canada* |
|  |  | Lack of perceived risk | *“A question I and my friends had in our mind [sic] was: How come some people smoke their whole lives and never have a problem?” (Poureslami et al, 2015)*  *Chinese in Canada* |
|  |  |  | *“Honestly, I don’t think there's anything bad with smoking – but my doctor told me to stop.” (Poureslami et al, 2015)*  *Chinese in Canada* |
|  |  |  | *“I feel the effects of smoking on my health but it is not enough to make me want to stop.” (Poureslami et al, 2015)*  *Chinese in Canada* |
|  |  | Beliefs (myths) about quitting smoking | *“I could stop breathing if I stopped smoking suddenly*.” *(Glasser et al, 2016)*  *Hispanic* |
|  |  | Did not enjoy rehab | *“I remember he had asked me for rehab twice. In one I went for 5-6 lessons; it was too boring. They said same thing over and over. I didn’t bother to go after that. That [rehab programme] was really boring, really boring.” (Early et al, 2020)*  *British Pakistani* |
|  |  | Exercise impossible | *“The exercises were not directly related to my lungs, they showed me exercises which was generally good for my body, there were all kinds of exercises and they taught me bits of all types of exercises. I would say they were excellent; the exercises are somewhat helpful. I have been asked to do them at home, I try to do them regularly but I am unable to do all the exercises the way they showed us in the classes.” (Early et al, 2020)*  *British Pakistani* |

**Table 3: Theme 3: Being able to access and attend care**

| **Synthesised themes (third order constructs)** | **Sub-themes** | **Second order constructs: the authors interpretations of the original findings** | **First Order constructs: examples of direct quotations from the participants of the study** |
| --- | --- | --- | --- |
| **Being able to access and attend care services** | Flexibility of attending the services | Rapport and flexibility of services | *“I did Wednesday and Friday. But then I couldn’t cope with Friday.”I did go, and I said, ‘I can’t do Fridays.” (Brighton et al, 2020)*  *Asian, Black, or Mixed* |
|  |  | Time of program | “I found that early morning was not good for me. I couldn’t concentrate on what they were doing. and the last thing I wanted to do was walk.” *(Levack et al, 2016)*  *Māori* |
|  |  |  | *“There’s plenty of time to catch the buses [to get to pulmonary rehabilitation]. Cause it – it didn’t start till half past ten O’clock.” (Levack et al, 2016)*  *Māori* |
|  |  | Duration of program | “Attending the class all the time, and it’s only an 8 weeks course, it’s not long enough for me though. I love it.” *(Levack et al, 2016)*  *Māori* |
|  | Transport and travel to pulmonary rehabilitation program | Transport & distance to program | *“I said to her “am I the only Maˉori coming?” And she said “well there’s meant to be others, but they’re not coming.” And I said “probably because you have to find your own way.” (Levack et al, 2016)*  *Maˉori* |
|  |  |  | *“I mean I know that going there might help but sometimes when you get out, when you can’t get out of bed or you can’t shower, it’s just I’m stuck to the oxygen. I have to stay home. I can’t do anything, that’s the only reason why I wouldn’t go. .” (Levack et al, 2016)*  *Māori* |
|  |  |  | *“Do you think you would be coming if it wasn’t for the [transport service] support? nah, I wouldn’t have been coming... Cause I can’t get around – well I can, but I don’t want to. You know, they pick me up and drop me off.” (Levack et al, 2016)*  *Māori* |
|  |  | Reliance on family for transport was a barrier reported by South Asian patients | *“If it’s far away, then I won’t be able to go, this is a problem. If it's in [Pxxx], then I can go. I drive.” (Early et al, 2020)*  *British Pakistani* |
|  |  |  | *“I only work 2-3 days. I don’t work a lot. Plus, I work at night, so I will be able to attend.” (Early et al, 2020)*  *British Pakistani* |
|  |  |  | *“If it’s in Luton, I can manage. If it is outside Luton, I will have to see because I don’t drive.” (Early et al, 2020)*  *British Pakistani* |
|  |  |  | *“It would have been very difficult [to attend if my daughter had not gone with me]…primarily [because of the] language. Although I can get a taxi myself, you have to sit there, they will tell you what you should do etc. It will be very difficult.” (Early et al, 2020)*  *British Pakistani* |
|  |  |  | *“That I can only be sure after I go, but I will try to complete the course. I was unsure who will take me there. But my daughter said she will. In my locality its once a week only.” (Early et al, 2020)*  *British Pakistani* |
|  | Opportunities for using digital technology or tele-services for care continuity | Access to PR right in one’s home | *The other thing I think it’s great, because in the home...winter time, it’s snowing, raining, sleet, and all that stuff... sometimes it’s not feasible to get there. So, if you got [the respiratory therapist] on the screen, you can still do what you got to do.” (Pekmezaris* *et al, 2020)*  *Hispanic or African American* |
|  |  | Safety and Comfort | *“You don’t have to struggle... with all the equipment, everything is set up just right for the patient to access everything.” (Pekmezaris* *et al, 2020)*  Hispanic or African American |
|  |  |  | *“The bike is not difficult, but the bike was breaking down. The arm wasn’t good... “. (Pekmezaris* *et al, 2020)*  *Hispanic or African American* |
|  |  | Convenience of being able to participate from home | *“I have a very difficult time walking.  I get out of breath very rapidly. So this was a godsend, or to me an excellent idea, in terms of doing it in-house. If I had to roll into a central location and do it, more than likely I would have not been in the program or else dropped out” (Polo et al, 2023)*  *African-American* |
|  |  | Learning from the RT how to perform physical and breathing exercises and medication management | “*He [the RT] also would show me via the tablet, how to use the medication.  You know, often you don't get to know how to do something very well, because the doctors often don't have the time to show you what to do with the medication they give you*” (*Polo et al, 2023)*  *Hispanic* |
|  |  | Feeling abandoned by the program and felt the program had been taken away from them. | *“They give the equipment in order to help you, and then they take it back from you. That breaks the morale of the whole program, you know. It’s like building you up and breaking you down” (Polo et al, 2023)*  *African-American* |

**Table4: Theme 4: The influence of communication and culture on a person’s care**

| **Synthesised themes (third order constructs)** | **Sub-themes** | **Second order constructs: the authors interpretations of the original findings** | **First Order constructs: examples of direct quotations from the participants of the study** |
| --- | --- | --- | --- |
| The influence of communication and culture on a person’s care | Language and interpretation to enable conversation and understanding | Unexpected need for interpreter | - *“Even I did not know much about it till you explained.* - *It is also possible that they did explain but I did not get it properly. They were in English.” (Early et al, 2020)*   *British Pakistani* |
|  |  | Communication through interpreters. | *“It’s about the language. I find that slightly difficult. But I can still make my doctor understand. They understand as well. Yes, I speak in English, they understand.” (Early et al, 2020)*  *British Pakistani* |
|  |  |  | *“I can understand everything, but I can’t say it. That is why I use an interpreter [at hospital]. I can speak, but at times there are some things that you are unable to say.” (Early et al, 2020)*  *British Pakistani* |
|  |  |  | *“I only had a little problem with English. My daughter in law accompanied me and sometimes my daughter would take me but mostly my daughter in law would go with me. She helped me with the paper work there as well.” (Early et al, 2020)*  *British Pakistani* |
|  |  |  | *“Although having someone helps but even if you went on your own one would learn by being there and watching them do the exercises and the things one didn’t understand they have everything explained on the paper /information leaflets. [The leaflets] were all in English but I do know that they have some in Urdu as well these days.” (Early et al, 2020)*  *British Pakistani* |
|  |  |  | *“I think this is fine [leaflets in English] as well as I said you can always get the children to read it for you.” (Early et al, 2020)*  *British Pakistani* |
|  |  |  | *“I could not understand [the education part] them. But I had my daughter with me she would explain things to me. I could not understand English.” (Early et al, 2020)*  *British Pakistani* |
|  |  |  | *“I think it’s largely because of language difficulty. If you don’t understand the language, you find it very difficult. People who speak the language can chat with others and joke while doing their exercise. They attend the classes happily.” (Early et al, 2020)*  *British Pakistani* |
|  |  | Information in patient's own language. | *“May be if [people] don’t understand they think attending will not benefit them.” (Early et al, 2020)*  *British Pakistani* |
|  |  |  | *“We are not very fluent in English. It is possible that this is why people do not go. I don’t understand 100%, but even I were to understand only 50% it is still beneficial. There is no harm.” (Early et al, 2020)*  *British Pakistani* |
|  |  |  | *“Yes, he [the son] would explain the English part to me. I don’t know much English. I have stopped speaking as well.* ***…****I don’t go out much. But I never knew lot of English, and I never spoke much in it. My children take me to the doctor. That will be very good [if the classes were in my own language]. It will be much easier for us to understand. Language is understandable. It’s not a big problem. I could understand whether it was right or left, up or down.” (Early et al, 2020)*  *British Pakistani* |
|  |  |  | *“But then in all your programmes…I saw that there were many Pakistani origin women who could hardly understand anything. Questions in English were put to them, and how would they answer when they did not understand a thing.” (Early et al, 2020)*  *British Pakistani* |
|  |  |  | *“There are so many diverse people that you can’t put an interpreter or translator for everyone. May be put some translations of lectures. I don’t know, I can’t tell you about that. It’s a hard thing to get so many…The problem of English is not only for Desi [people from Subcontinent], Indians and Pakistanis, there are Arabs, now there are Polish. So, I don’t know how many people you are going to employ there.” (Early et al, 2020)*  *British Pakistani* |
|  |  |  | *“You know people there are mostly elderly. Young people are mostly conversant in English. For elderly I said that if there are people like you around who would debrief them after every 10 minutes, it will make them more interested. Otherwise, it will be like dancing in front of the blind. They can hear you but can’t make sense.” (Early et al, 2020)*  *British Pakistani* |
|  |  | Connection with things Maˉori was also considered highly important when first receiving information about pulmonary rehabilitation programs | *“[Talking about the hospital brochure on pulmonary reha- bilitation:] There’s nothing that says “if you are Maˉori, this is what you need.” It says you take part. And I was looking for the Maˉori side. And then I came here [the marae] and this was all Maˉori, and in the Maˉori environment, it – it was – for me, I thought oh, it must work. It has to.” (Levack et al, 2016)*  *Māori* |
|  | Feelings of belonging within a community | Locating the program at a marae also inherently connected these participants to their wider community | *“And it just that whanaungatanga [connecting with others] time is very important, how everyone feels. It’s something personal to yourself. But some get to say more than others... But it’s a kind of down to our level, and it’s good to bringing the tikanga aspect side of things, tikanga Maˉori, our waiata, how we do things, who with, and in a place that we feel good in being.” (Levack et al, 2016)*  *Māori* |
|  |  | Considerable value was placed on the time that was spent after the formal class session on sharing stories with one another and with the program staff | *“The tikanga [protocol] aspect of it – we talk about our whakapapa [genealogy] first, that’s all part of whanaungatanga [connection between people], and our stories are very important. Our stories are very important... When you come to the culture side of thing, you break that down even more to simple waiata, whakapapa, and te reo Maˉori, all that is our wellbeing. It’s not just the having to learn what the Paˉkehaˉ tell us to do.” (Levack et al, 2016)*  *Māori* |
|  |  | Time for whakawhanaungatanga | *“The lady [a Maˉori nurse] who picks us up [for pulmonary rehabilitation] ... She asked me the question when she saw it [a non-invasive nasal ventilation machine for sleep apnea in the participant’s home], and she said “do you use that?” And – and I looked at her. And she goes “no, you don’t aye?” I went “no.”... She happened to see it sitting there, and she asked me...*  *Interviewer: So you hadn’t told anyone else you weren’t using it?*  *No, I just kept quiet about it. I didn’t know you had to come to the hospital after, and they check your machine, you see... So now I just felt relaxed with her. And yeah, I just – every question she asked I just answered it to my best knowledge.” (Levack et al, 2016)*  *Māori* |
|  | Appreciating and representing cultures | Culture | *“We are mostly less educated people, and we are shy, that is why I think our people may not be attending these programmes. I think it’s a very beneficial programme where you get help for free. Thank God that we are here and we get these opportunities. There is no discrimination based on anything when it comes to treatment. They treat everyone the same. This is very good.” (Early et al, 2020)*  *British Pakistani* |
|  |  | Culturally Tailoring the Recruitment Process | *“...the video was very good, but I think it will be much better when they get more people involved...they’re communicating together at the same time, it’s like...supporting each other, you know...*  *It didn’t impress me much. I liked the part that you played, I thought you did a very good job there. But he doesn’t really look the part...*  *I think you want to think about incorporating the caregiver, because...it’s not just the patient... it’s the spouse (Mrs Lopez)( *names changed to protect patient privacy) who’s been watching... been part of the story of Mr. Lopez, just as much as he’s been a part. Different role, but together. [Mrs. Lopez] was very clear and very eloquent talking about this. And... [Mrs Lopez] said that now you can’t get him off the bike... It’s an inspiration to see Mr. Lopez, but also to hear Mrs. Lopez...So I would just suggest you think about the caregiver.” (Pekmezaris et al, 2020)*  *Hispanic or African American* |
|  |  | Gender | *“I think it will be a bit difficult for women. Because the culture of our women is different. The gathering is mixed. I think they may not be very happy attending. If the gathering has other women of their culture, then it may become easier for them.” (Early et al, 2020)*  *British Pakistani* |
|  |  |  | *“I did not like this [mixed classes]. Men should be separate from women. This was the most difficult part.” (Early et al, 2020)*  *British Pakistani* |
